# Tailoring Prevention and Control Strategies for Childhood Tuberculosis: From a Global Analysis of Burden Trends and Inequalities Across Three Age Groups (1990–2021) to Prevention and Control Strategies

**DOI:** 10.3390/tropicalmed11050129

**Published:** 2026-05-09

**Authors:** Xiaoming Liu, Howard Takiff, Hui Jiang, Weimin Li

**Affiliations:** 1 Beijing Chest Hospital, Capital Medical University, Beijing 101149, China; liuxiaoming313@163.com; 2 Beijing Tuberculosis and Thoracic Tumor Research Institute, Beijing 101149, China; 3 Instituto Venezolano de Investigaciones Científicas, Centro de Microbiología y Biología Celular, Caracas 1020A, Venezuela

**Keywords:** childhood tuberculosis, incidence, mortality, socio-demographic index, Bayesian age-period-cohort model

## Abstract

**Background:** Childhood tuberculosis (TB) is a major but underappreciated threat to human health. Because diagnosis of tuberculosis in children is difficult, there are a lack of accurate global statistics. This study aimed to comprehensively assess the long-term global, regional, and age-specific burden of childhood TB from 1990 to 2021, to examine its temporal trends and socioeconomic inequalities, and to project future patterns through 2045. **Methods:** We used incidence and mortality data from the GBD 2021 database for TB in children ages 0–14 years from 1990 to 2021. Children were stratified into three age groups—<5, 5–9 and 10–14 years—and classified by region and Socio-Demographic Index (SDI). Multiple statistical approaches were employed, including average annual percentage change and Bayesian age-period-cohort models, to analyze spatiotemporal trends in disease burden and generate projections for the next 20 years. We used decomposition analysis to separate demographic from epidemiological drivers and concentration indices to quantify socioeconomic inequalities. **Results:** In 2021 there were, globally, an estimated 759,300 incident cases of childhood TB and 70,659 deaths. Since 1990, childhood TB incidence and mortality rates have declined at average annual rates of 2.61% and 4.48%, respectively. The SDI showed a significant negative correlation with both incidence and mortality of childhood TB (*p* < 0.05). In 2021, 78.01% of childhood TB deaths were in children under 5 years of age, and over 80% of global childhood TB deaths occurred in Sub-Saharan Africa. Epidemiological interventions were partly offset by rapid population growth in low-SDI regions. The trends show that the incidence and mortality will continue to decline through 2045, but not enough to meet the goal of eliminating childhood TB by 2035. **Conclusions:** Global efforts should adopt an age-specific framework that prioritizes universal preventive treatment to eliminate mortality in children under 5 years, and implements active case finding to reduce transmission chains among children 5–14 years. Sustaining the decrease in the TB burdens of low-SDI regions requires international financing strategies attuned to expanding populations to ensure epidemiological success is not erased by demographic growth.

## 1. Introduction

Tuberculosis (TB) remains a major global public health challenge [1,2,3]. Each year, more than one million people die from TB worldwide, and approximately 12% of these deaths occur in children younger than 15 years, indicating a substantial pediatric disease burden [4,5,6]. A South African birth cohort study revealed intense exposure to *Mycobacterium tuberculosis* during the first decade of life, with approximately 36% of children showing evidence of having been infected with TB and 10% developing TB disease [7,8]. However, due to the unique pathophysiological characteristics of childhood TB and the difficulty in diagnosing TB in children [9,10], only about 18% of childhood TB cases were officially notified in 2013 [11,12]. Since 2015, estimates of the global TB disease burden based on Global Burden of Disease (GBD) data have calculated 690,262 cases of childhood TB annually [13]. However, using mathematical modeling, Menzies and colleagues [14] estimated childhood TB cases at 997,500 in 2019. That same year, the WHO estimated a total of 1 million cases global childhood TB cases, of which only approximately 520,000 were reported, yielding an underreporting rate of 48% [4,15].

Previous GBD-based studies have focused primarily on descriptive trends of overall TB incidence and mortality, often overlooking distinct biological and epidemiological nuances [16]. Although the GBD database provides foundational evidence for TB disease burden research [17], treating childhood TB (0–14 years) as a single entity in policy planning obscures critical heterogeneities. Biologically, the risk profile changes dramatically with age. Children under 5 years are prone to severe, disseminated disease (e.g., meningitis, miliary TB) with high mortality [18], while adolescents (10–14 years) may present with adult-type pulmonary disease, sometimes including cavitary forms [19]. Existing aggregate estimates fail to recognize these distinct clinical tendencies, potentially leading to inefficient resource allocation.

Although global TB rates are declining, these gains may be compromised by rapid population growth in regions with a low Socio-Demographic Index (SDI) and the persistent inequality gap between rich and poor nations. It is well established that childhood TB incidence and mortality demonstrate a negative correlation with the SDI [20].

Therefore, assessing childhood TB risk across three age groups (<5, 5–9, and 10–14 years) at different SDI levels may provide more robust evidence for targeted prevention and control strategies. To analyze the many factors determining overall trends in childhood TB, this study used EAPC, Joinpoint regression, BAPC, APC, health inequality analysis, and decomposition analysis to examine temporal trends, age and period effects, socioeconomic disparities, demographic contributions and to predict future trends. Based on these analyses, the study sought to: (1) identify age-specific intervention targets; (2) quantify the demographic drivers that hinder control efforts; and (3) highlight the socioeconomic inequalities that future strategies must address.

## 2. Methods

### 2.1. Study Population and Data Sources

#### 2.1.1. Data Sources

TB burden data from 1990 to 2021 were obtained from the GBD 2021 through the Global Health Data Exchange (GHDx) tool (Institute of Health Metrics and Evaluation (IHME)) GBD Results; 2021 (https://vizhub.healthdata.org/gbd-results/, accessed 15 July 2024). The GBD estimates are derived from multiple underlying data sources, including vital registration systems, mortality surveillance systems, national disease notification data, household surveys, censuses, and published epidemiological studies. Consequently, this study represents a secondary analysis utilizing GBD’s modeled estimates for childhood TB (0–14 years), rather than primary individual-level patient databases. Detailed methodologies for GBD data and TB estimation have been published previously [20,21].

#### 2.1.2. Socio-Demographic Index

SDI is a composite indicator measuring socioeconomic status, demographic structure, and development level across countries and regions. The SDI estimates are based on methodologies described in recent GBD studies [22,23,24].

#### 2.1.3. Childhood TB and Disease Burden

Childhood tuberculosis was identified according to the GBD 2021 cause definition for tuberculosis, corresponding to ICD-10 codes A15–A19 and B90 and ICD-9 codes 010–018 and 137. These estimates include both bacteriologically confirmed cases and clinically diagnosed unconfirmed cases. The study population comprised children under 15 years of age, stratified into three age groups: <5, 5–9, and 10–14 years [25]. Incidence, mortality, and their corresponding age-standardized rates (ASR) and SDI data were downloaded from GBD 2021.

### 2.2. Trend Analysis

#### 2.2.1. Estimated Annual Percentage Change (EAPC) Model

EAPC is a metric used for assessing temporal changes in age-standardized rates. It was estimated using the log-linear regression model ln (ASR) = α + β × year + ε, where ASR represents the age-standardized rate, α is the intercept, β is the slope, and ε is the error term. The EAPC was calculated as 100 × [exp(β) − 1] [26]. We calculated EAPC using least-squares linear regression models, with results organized and analyzed using the broom package [26]. This method allows us to summarize the long-term trends in childhood TB burden.

#### 2.2.2. Joinpoint Regression Analysis

The Joinpoint regression model is a piecewise linear regression method used to quantify temporal trends in epidemiological indicators such as age-standardized incidence and mortality rates [27]. Joinpoint regression analysis was performed using the Joinpoint Regression Program (version 5.2.0.0), with up to 5 joinpoints allowed. Candidate joinpoints were identified using the Grid Search Method (GSM), and the optimal model was selected using the Monte Carlo Permutation Test. This approach enabled us to detect changes in the temporal trends of childhood TB burden.

### 2.3. Projections

#### Bayesian Age-Period-Cohort (BAPC) Model

The BAPC model was employed to project future incidence and mortality rates of childhood TB. The BAPC model addresses parameter estimation challenges arising from the linear relationships among age, period, and cohort in traditional APC models by incorporating Bayesian priors and utilizing the Integrated Nested Laplace Approximation (INLA) algorithm [28,29]. To ensure projection accuracy and robustness, we utilized childhood TB incidence data from 1990 to 2021 to forecast incidence rates from 2022 to 2045. This method provides valuable insights into potential future trends in childhood TB under different scenarios.

### 2.4. Risk Factors

#### 2.4.1. Age-Period-Cohort (APC) Model

The APC model was used to analyze and estimate the effects of age, time period, and birth cohort on childhood TB burden. By decomposing these effects, the APC model facilitates deeper exploration of underlying mechanisms influencing TB incidence and prevalence [30]. This method contributes to a better understanding of how the burden of childhood tuberculosis varies across age groups and changes over time.

#### 2.4.2. Decomposition Analysis

Decomposition analysis was used to identify the primary factors underlying changes in the burden of childhood TB from 1990 to 2021. This approach quantifies the independent contributions of population growth, population aging. and epidemiological changes while holding other factors constant [31,32]. It provides valuable insights into how demographic changes and epidemiological factors have influenced childhood TB incidence and mortality over time.

#### 2.4.3. Health Inequality Analysis

Cross-national health inequality analysis measured the absolute and relative inequality in childhood TB burden distribution by calculating the Slope Index of Inequality (SII) and the Erreygers-corrected concentration index (ECI). To better control for bias and heterogeneity, we employed robust linear regression models rather than ordinary linear regression models in the health inequality analysis [33]. Robust regression models reduce sensitivity to outliers, minimize bias induced by data heterogeneity or extreme values, and more accurately represent health inequalities. This approach can better reflect socioeconomic disparities in the burden of tuberculosis.

### 2.5. Ethics Statement

This study involves secondary analysis of the GBD database. As the data are aggregated, de-identified, and publicly available, institutional review board (IRB) approval and patient informed consent were not required.

### 2.6. Patient and Public Involvement Statement

It was not appropriate or possible to involve patients or the public in the design, conduct, reporting or dissemination plans of our research, as this study relied exclusively on existing secondary data.

## 3. Results

### 3.1. Global Burden of Childhood TB in 2021

In 2021, an estimated 759,300 incident cases of childhood TB (95% uncertainty interval [UI]: 528,472.7–1,051,892.4) occurred globally, corresponding to an age-standardized incidence rate (ASIR) of 38.1 per 100,000 (95% UI: 26.7–52.6). During the same year, childhood TB accounted for 70,659 deaths (95% UI: 53,028.3–89,634.3), with an age-standardized mortality rate (ASMR) of 3.7 per 100,000 (95% UI: 2.8–4.7).

**Figure 1:**
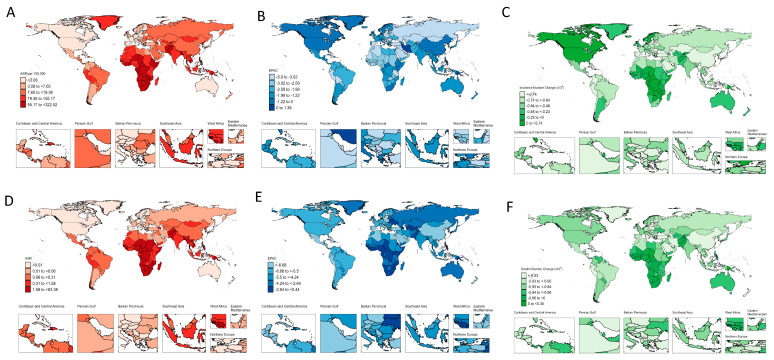
Global Distribution of childhood tuberculosis burden (1990–2021). (**A**) Age-standardized incidence rate (ASIR) per 100,000 population. (**B**) Estimated annual percentage change (EAPC) in incidence. (**C**) Change in incidence number (absolute change). (**D**) Age-standardized mortality rate (ASMR) per 100,000 population. (**E**) Estimated annual percentage change (EAPC) in mortality. (**F**) Change in mortality number (absolute change).

Compared with 1990, all indicators of global childhood TB burden showed sustained declines. The EAPC in ASIR was −2.61% (95% UI: −2.74 to −2.48), and the EAPC in ASMR was −4.48% (95% UI: −4.71 to −4.24) (Table 1, Figure 1, Table S1).

### 3.2. Socio-Demographic Index and Regional Heterogeneity in Disease Burden

Substantial socioeconomic and geographic disparities in childhood TB burden persisted between 1990 and 2021, despite declines across all SDI regions. In 2021, the low-SDI region had the highest incidence burden, with an ASIR of 69.7 per 100,000 (95% UI: 49.2–96.2), whereas the high-SDI region had the lowest incidence burden, with an ASIR of 2.1 per 100,000 (95% UI: 1.4–3.1) (Table 1).

Regional temporal trends were heterogeneous. The high-income Asia Pacific region showed the largest decline in incidence (EAPC = −5.63, 95% UI: −5.79 to −5.47), whereas Oceania was the only region with an increasing incidence trend (EAPC = 0.15, 95% UI: 0.10–0.21). Mortality burden was highest in Sub-Saharan Africa, particularly Central Sub-Saharan Africa, where the ASMR reached 14.8 per 100,000 (95% UI: 9.2–23.3) (Table 1, Figure 1).

At the country level, Equatorial Guinea showed the largest increase in incidence (EAPC = 2.53, 95% UI: 2.39–2.67), whereas Brazil showed the largest decline (EAPC = −1.57, 95% UI: −1.73 to −1.41) (Table S1, Figure 1).

### 3.3. Long-Term Trends and Future Projections

**Figure 2:**
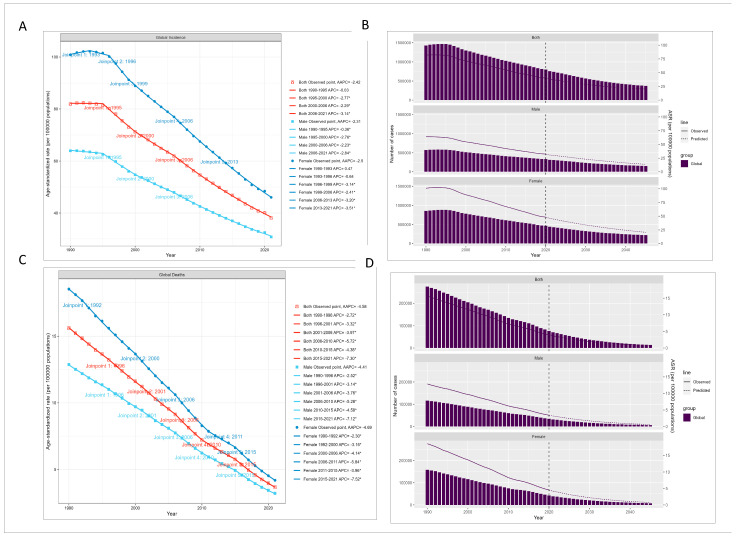
Trends in childhood tuberculosis incidence and mortality based on Joinpoint and BAPC models. (**A**). Global childhood tuberculosis incidence trends: Joinpoint regression analysis showing changes in ASIR from 1990 to 2021, with projections through 2045 by sex and age group. (**B**). Childhood tuberculosis incidence projections: BAPC model projections for global incidence trends from 2021 to 2045, with observed and predicted cases for both sexes. (**C**). Global childhood tuberculosis mortality trends: Joinpoint regression analysis of ASMR from 1990 to 2021, with future projections. (**D**). Childhood tuberculosis mortality projections: BAPC model projections for global childhood TB deaths, observed and predicted trends from 2021 to 2045.

Joinpoint regression analysis showed significant declining trends in all major indicators of global childhood TB burden from 1990 to 2021 (all AAPC *p* < 0.05). The average annual percent change was −2.42% for incidence and −4.58% for mortality. A significant joinpoint for incidence was identified during 1990–1995 (Figure 2).

Projections based on the Bayesian age-period-cohort (BAPC) model indicated continuing declines in both incidence and mortality through 2045, with a greater reduction in mortality than in incidence. However, under the projected trajectories, the goal of ending the childhood TB epidemic by 2035 [34] was not reached (Figure 2).

### 3.4. Age-Period-Cohort Effects and Decomposition of Driving Factors

Age-period-cohort analysis showed variation in childhood TB burden across age groups, periods, and birth cohorts. The <5 years age group had the highest annual rate of change in disease burden, whereas the 10–14 years age group had the lowest. Period effects peaked during 1992–1996 and declined thereafter, reaching their lowest levels in 2017–2021. Cohort effects showed that children born during 1986–1997 had the highest estimated risk, whereas those born during 2012–2021 had the lowest estimated risk (Figure S1).

**Figure 3:**
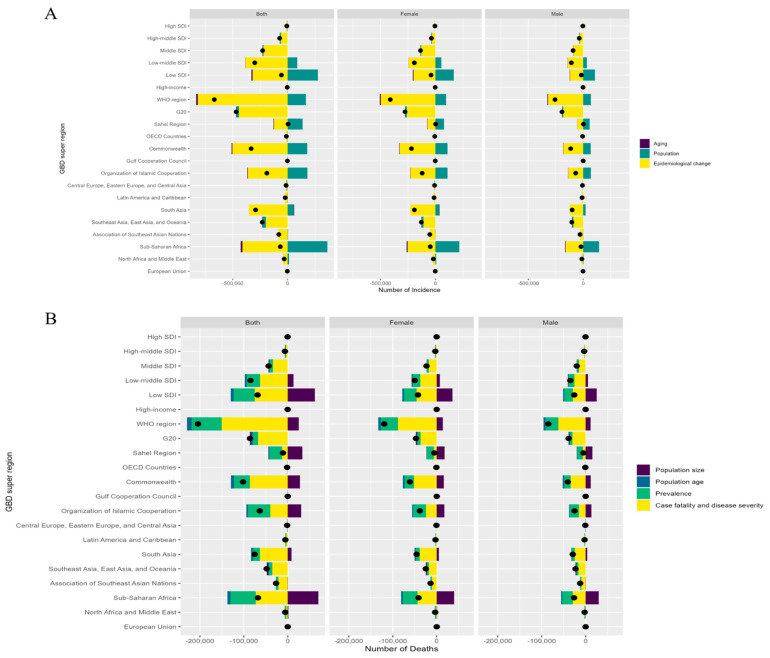
Decomposition analysis of childhood tuberculosis incidence and mortality. (**A**). Decomposition of childhood tuberculosis incidence: the contributions of population growth, aging, and epidemiological shifts to changes in incidence from 1990 to 2021. (**B**). Decomposition of childhood tuberculosis mortality: the contributions of population growth, aging, and case fatality to changes in mortality from 1990 to 2021.

Decomposition analysis showed that population growth contributed to the absolute increases in both incident cases and deaths. With respect to incidence, favorable changes in epidemiological factors and population ageing both acted to reduce the rate, whereas the decline in mortality was primarily attributable to reductions in case fatality and prevalence (Figure 3).

### 3.5. Socioeconomic Determinants and Health Inequalities

**Figure 4:**
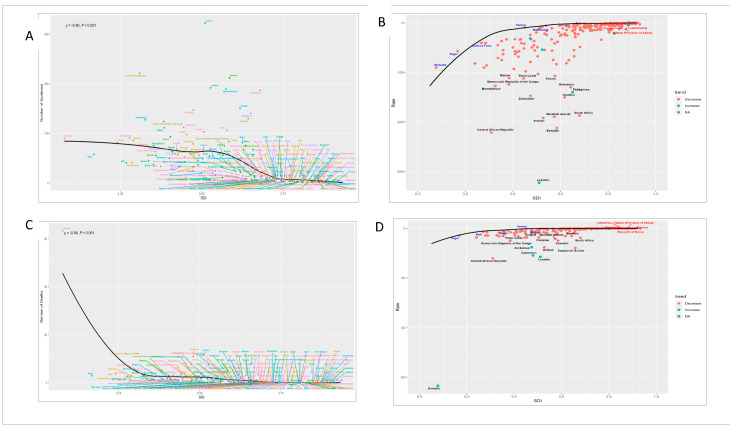
Correlation between SDI and childhood tuberculosis burden across countries. (**A**). Childhood tuberculosis incidence number vs. SDI. (**B**). Childhood tuberculosis incidence rate vs. SDI: frontier analysis. (**C**). Childhood tuberculosis mortality number vs. SDI. (**D**). Childhood tuberculosis mortality rate vs. SDI: frontier analysis.

**Figure 5:**
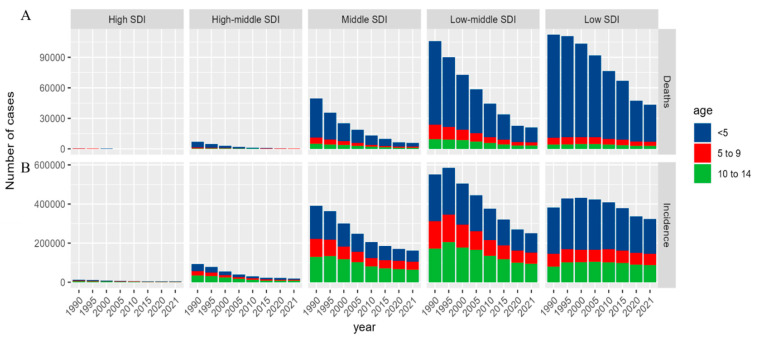
Temporal trends in childhood tuberculosis incidence and mortality by SDI group and age category (1990–2021). (**A**) Number of deaths; (**B**) number of incident cases.

The burden of childhood TB varied substantially across SDI regions. In low-SDI regions, deaths among children younger than 5 years remained markedly higher than in other SDI strata, peaking at approximately 90,000 before declining to approximately 60,000 by 2021. In contrast, high-SDI regions consistently recorded very low numbers of deaths across all age groups throughout the entire study period (Figure 4 and Figure 5).

Among children younger than 5 years, the number of cases was also substantially higher in low-SDI regions than in high-SDI regions. After 2015, both incidence and mortality declined across all SDI strata, as shown in Figure 5, although the magnitude of decline varied by region. The decrease in mortality was most apparent in the high–middle SDI region, while incidence declined most visibly in the lower SDI strata, particularly the low–middle and low-SDI regions. Country-level analyses further showed heterogeneous patterns. Lesotho, the Philippines and Liberia experienced sustained increases in incidence, whereas Lesotho, Zimbabwe, and Cameroon showed continued increases in mortality. In contrast, the Central African Republic, Kiribati and Equatorial Guinea showed declining mortality (Figure 4A–D).

Health inequality analyses showed that inequality in incidence decreased over time, whereas mortality inequality remained pronounced. The concentration index for incidence improved from −0.32 in 1990 to −0.32 in 2021 (95% CI: −0.37 to −0.26), while the concentration index for mortality in 2021 was −0.42 (95% CI: −0.49 to −0.36). Cumulative distribution curves showed that more than 60% of childhood TB cases were concentrated in countries ranked in the bottom 40% of the SDI distribution (Figure S2).

## 4. Discussion

This study, based on GBD data, estimated that 759,000 childhood TB cases occurred globally in 2021, with an ASIR of 38.1 cases per 100,000 population, declining from 1990 at an average annual rate of 2.61%. Childhood TB deaths in 2021 were estimated at 70,700, with an ASMR of 3.7 per 100,000, demonstrating a more pronounced average annual decline of 4.48%. The SDI showed a significant inverse correlation with both incidence and mortality of childhood TB (*p* < 0.05), leading to marked regional disparities. Sub-Saharan Africa, a low-SDI region, bore more than 80% of global childhood TB deaths, with 78.01% in children younger than 5 years. Model projections indicate that both incidence and mortality will continue to decline through 2045, although these declines will be insufficient to achieve the goal of ending the childhood TB epidemic by 2035.

Childhood TB has been substantially underestimated globally for decades [35,36]. Our estimate of 759,000 global childhood TB cases in 2021 aligns closely with previous findings [16]. Yerramsetti et al. have argued that WHO overestimated the global childhood TB burden, whereas GBD data underestimated the burden [14]. Because our analyses were based on GBD data, our results may also be underestimations. With advances in technologies for diagnosing TB in children, case-detection rates should improve, and therefore estimates of the TB burden should become increasingly accurate.

Our finding that 78.01% of childhood TB deaths occurred among children younger than 5 years confirms this age group as an extremely high-risk population for tuberculosis-related mortality. WHO therefore strongly recommends TB preventive treatment for household contacts younger than 5 years [37], and Malik et al. have proposed a preventive 1-month daily regimen of rifampicin and isoniazid [38]. Martinez et al. have suggested that in low-burden settings, preventive treatment should prioritize all contacts <5 years of age and older contacts with confirmed evidence of infection, whereas in high-burden settings, preventive treatment should be given to all contacts [7]. Although this real-world trend aligns with GBD findings in our study, substantial differences in absolute incidence rates highlight the critical importance of conducting high-quality active case-finding studies in middle and high-SDI countries to obtain more accurate childhood TB epidemiological data.

Decomposition analysis quantified the demographic factors shaping the burden of childhood TB and suggested that improvements in TB control and disease management were likely the main drivers for the declines in incidence and mortality. Although population growth increased the absolute numbers of both incident cases and deaths, advances in screening, diagnosis and treatment contributed to reduced case fatality. The use of highly accurate diagnostic tools such as Xpert Ultra [39], along with testing of non-traditional specimens such as stool and nasopharyngeal aspirates, has improved the diagnostic capacity for childhood TB. The reduction in mortality may also reflect broader public health and clinical advances, including expanded antiretroviral therapy for children with TB/HIV co-infection, improved management of malnutrition and severe disease, and the scale-up of child-focused TB care in high-burden settings. However, even with the improved diagnostic tests, only 52% of global childhood TB cases are currently detected, leaving an urgent need for more sensitive diagnostic technologies and wider implementation of testing, particularly in low-SDI regions [40]. This finding is consistent with the results of the decomposition, inequality, and SDI analyses, indicating that current improvements in childhood TB control remain unevenly distributed across socioeconomic settings. When combined with the projection results, it further suggests that although the current declining trends are likely to continue, they will still be insufficient to achieve the 2035 targets of the WHO End TB Strategy, particularly among children.

Our findings revealed that more than 60% of childhood TB cases are concentrated in countries ranking in the lowest 40th percentile of SDI, with sub-Saharan Africa—a low-SDI region—accounting for over 80% of global childhood TB deaths (Figure 1). The inverse association between SDI and childhood TB burden is well established, and likely the result of limited resources leading to weak primary healthcare, high HIV prevalence, undernutrition, limited access to pediatric TB diagnostic tools, delayed case detection and constrained health system capacity. Although factors such as malnutrition and Bacillus Calmette-Guérin (BCG) vaccination coverage are closely associated with childhood TB mortality, high-quality clinical treatment capacity remains the critical determinant of mortality reduction [41]. In low-SDI regions, the treatment of childhood TB is heavily dependent upon international funding [42], which, unfortunately, has been recently curtailed. Menzies and colleagues have estimated that discontinuation of US bilateral health aid and Global Fund programs during 2025–2034 would result in an additional 2.5 million childhood TB cases and 340,000 deaths. Broader weakening of Global Fund support could lead to 8.9 million additional cases and 1.5 million deaths, effectively doubling the current TB burden, with Africa and southeast Asia the most severely affected [43].

In line with our findings that population growth offsets part of the epidemiological gains and that mortality remains concentrated in low-SDI regions, further reductions in the incidence and mortality of childhood TB will require health assistance beyond the baseline 2025 levels in low-SDI regions, particularly Africa and southeast Asia. However, priorities are unlikely to be uniform across regions. In Africa, strengthening primary care capacity, household contact investigation, and integration of childhood TB screening into routine child health services may be especially important. In southeast Asia, where under-detection and delayed diagnosis remain major barriers, priority should be given to active case finding, improved referral pathways and wider access to rapid molecular testing. The health inequality and SDI correlation analyses further suggest that persistent inequality in childhood TB remains concentrated in several high-burden countries, including Lesotho, Zimbabwe, Cameroon, Liberia, and the Philippines, where the burden remains disproportionately high, despite some improvement in incidence-related inequality. Overall, incidence-related inequality showed some narrowing over time, whereas mortality-related inequality remained persistent, indicating that reductions in childhood TB deaths have been less equitable than reductions in incidence. In these settings, implementation priorities should include systematic screening and preventive management of household contacts aged <5 years, timely diagnostic testing, prompt treatment initiation, and strengthened follow-up through primary care systems.

This study possesses several notable strengths. First, it provides a comprehensive and up-to-date global assessment of childhood TB burden across age groups using standardized GBD methodology, thereby enabling direct comparisons across 204 countries and territories over a 32-year period. Importantly, this study integrates inequality analysis, decomposition analysis, and projection modelling to examine demographic drivers, socioeconomic inequalities, and future trends in childhood TB within a single analytical framework, thereby providing a more comprehensive understanding of age-specific burden patterns and their underlying drivers. Second, the application of multiple analytical methods—including Joinpoint regression, BAPC modelling, and decomposition analysis—allowed us to identify temporal inflection points, disentangle age-period-cohort effects, and quantify the relative contributions of demographic versus epidemiological factors to trends in childhood TB. Third, our systematic examination of health inequalities through concentration and slope indices provides robust evidence of persistent socioeconomic gradients in childhood TB, thereby showing where targeted intervention strategies could have significant effects. Fourth, the projections through 2045 offer useful guidance for long-term planning, while highlighting that current rates of decline are insufficient to achieve the goal of eliminating childhood TB by 2035.

This study has several limitations. First, all burden estimates were derived from the GBD 2021 framework and are therefore subject to model-based uncertainty, particularly in settings with sparse or variable-quality surveillance data. These estimates should be interpreted as modelled approximations rather than precise counts. Second, childhood TB remains substantially underdiagnosed and underreported because of its non-specific clinical presentation, difficulties in microbiological confirmation, and limited access to sensitive, child-friendly diagnostic tools. This diagnostic gap inevitably leads to underestimation of the true burden of childhood TB, especially in routine surveillance systems. Third, these limitations are likely to be more pronounced in low-SDI settings, where restricted diagnostic access, weaker surveillance systems and inadequate case-detection capacity may introduce additional bias. In addition, our analysis could not capture subnational heterogeneity within countries, potentially masking important within-country disparities. Finally, these BAPC projections should be interpreted as conditional forecasts based on historical case-detection patterns rather than direct evidence of future transmission dynamics. Because the models assume continuity of past trends and do not capture major disruptions such as COVID-19, funding instability or drug resistance, the projected declines should not be read deterministically. This is especially important in low-SDI settings, where future, improved diagnostic methods may temporarily increase the notified incidence of childhood TB by detecting a greater percentage of cases than can be diagnosed with currently available techniques.

## 5. Conclusions

Advances in diagnostic and therapeutic technologies have been the primary drivers of the decline in the global childhood TB burden, yet demographic expansion and deep-seated socioeconomic inequalities threaten to stall this progress. The era of treating childhood TB as a single entity must end. Future global efforts should prioritize universal preventive treatment to reduce TB mortality in children <5 and active case finding to cut transmission chains among adolescents. Sustaining the reductions in childhood TB incidence and mortality in low-SDI regions requires international financing strategies that are indexed to population growth, thereby ensuring that hard-won epidemiological successes are not erased by demographic realities.

## Figures and Tables

**Table 1:** Global and regional trends in childhood tuberculosis burden: incidence and mortality (1990–2021).

Location	1990		2021		EAPC_95%CI
	Number (95%UI)	ASR (95%UI)	Number (95%UI)	ASR (95%UI)	
**Incidence**					
Global	1,428,395.6 (999,118.2, 1,957,605.2)	81.9 (57.2, 112.4)	759,300.5 (528,472.7, 1,051,892.4)	38.1 (26.7, 52.6)	−2.61 (−2.74, −2.48)
High SDI	12,248 (8501.9, 17,141.4)	6.6 (4.6, 9.2)	3724.6 (2453.3, 5467.3)	2.1 (1.4, 3.1)	−3.71 (−3.88, −3.53)
High–middle SDI	92,430 (63,618.3, 128,910.7)	33.7 (23.3, 47)	19,078.8 (13,096.7, 27,310)	8.3 (5.8, 11.8)	−4.59 (−4.7, −4.49)
Middle SDI	390,644.9 (271,179, 533,431.9)	67.7 (47, 92.4)	161,888.3 (111,802.7, 223,935)	28.7 (20, 39.4)	−2.9 (−2.97, −2.82)
Low–middle SDI	551,050.5 (373,992.7, 767,685.7)	116.6 (78.8, 162.8)	250,777.8 (171,583.3, 349,520.1)	43.3 (29.8, 60.1)	−3.42 (−3.57, −3.28)
Low SDI	381,303.3 (275,933.8, 510,755.9)	160.2 (114.7, 216.1)	323,362.9 (228,552.6, 445,156.3)	69.7 (49.2, 96.2)	−2.82 (−2.99, −2.66)
High-income North America	487.4 (306.7, 744.6)	0.8 (0.5, 1.2)	518 (334, 770.7)	0.8 (0.5, 1.2)	1.21 (0.7, 1.72)
Andean Latin America	12,660.8 (8695.9, 18,034.6)	85.2 (58.5, 121.5)	3165.3 (2145.6, 4493.5)	17.4 (11.8, 24.7)	−5.63 (−5.96, −5.3)
Tropical Latin America	8299.8 (5560.9, 11,763.3)	15.7 (10.6, 22.1)	3017.6 (1981.4, 4351.8)	6 (3.9, 8.6)	−4.57 (−5.27, −3.87)
Central Latin America	8683.7 (6076.9, 11,865.8)	13.4 (9.4, 18.4)	2666.4 (1810, 3840.6)	4.2 (2.9, 6)	−4.39 (−4.68, −4.11)
Southern Latin America	1617.4 (1068.9, 2342.1)	10.8 (7.2, 15.6)	740.5 (474, 1072.5)	5 (3.2, 7.2)	−2.85 (−3.05, −2.65)
Western Europe	3971.4 (2603.2, 5760.9)	5.5 (3.6, 8)	1740.7 (1086.7, 2650.3)	2.5 (1.6, 3.8)	−2.12 (−2.32, −1.91)
Central Europe	3252.2 (2164.2, 4720.9)	11 (7.4, 15.9)	677.5 (450.2, 967)	3.8 (2.5, 5.4)	−3.38 (−3.42, −3.34)
Eastern Europe	12,179.4 (7834.2, 18,183.3)	23.6 (15.2, 35.1)	3430.1 (2112.7, 5121.4)	9.3 (5.8, 13.8)	−1.48 (−1.94, −1.01)
High-income Asia Pacific	1773.6 (1268.3, 2385.9)	5 (3.6, 6.6)	190.8 (119.9, 285.7)	0.8 (0.5, 1.2)	−5.63 (−5.79, −5.47)
Central Asia	8286.2 (5710.5, 11,635.2)	32.9 (22.5, 46.5)	4086.6 (2848.2, 5633.6)	14.8 (10.3, 20.5)	−2.11 (−2.43, −1.79)
South Asia	487,209.5 (313,278.8, 701,510.4)	112.8 (72.3, 162.8)	194,113.4 (131,224.6, 272,746.5)	38 (25.9, 53.1)	−3.94 (−4.1, −3.78)
Southeast Asia	179,068.5 (124,278.9, 247,996.6)	104.9 (72.9, 145.1)	96,961.5 (67,198.4, 134,478.7)	56.1 (39.1, 77.5)	−2.18 (−2.44, −1.92)
East Asia	178,503 (121,626.5, 246,860.3)	53.9 (36.7, 74.6)	27575 (19,393.3, 38,464.5)	10.6 (7.6, 14.7)	−5.34 (−5.5, −5.19)
North Africa and Middle East	54,427 (38,752.4, 74,697.4)	38.8 (27.5, 53.4)	22,026.6 (14,916.8, 31,397.8)	12 (8.2, 17.1)	−3.59 (−3.87, −3.32)
Western Sub-Saharan Africa	156,226.7 (116,124, 205,636.1)	166.1 (122, 220.8)	131,996.7 (94,441.7, 178,611.2)	60.3 (42.9, 81.9)	−3.26 (−3.52, −3)
Eastern Sub-Saharan Africa	163,903.8 (121,045.6, 216,567.8)	172.3 (125.9, 229.5)	136,955.1 (92,785.1, 191,759.1)	76.3 (51.6, 107)	−2.92 (−3.09, −2.75)
Central Sub-Saharan Africa	73,028.2 (52,212.6, 99,209)	274.2 (193.6, 376.3)	85,204.8 (60,620.5, 116,413.7)	144.3 (102.4, 197.5)	−2.06 (−2.33, −1.8)
Southern Sub-Saharan Africa	68,464.2 (49,092, 90,625.1)	327.9 (234.7, 434.7)	39,541.5 (25,939.7, 55,506.3)	164.9 (108.8, 230.8)	−1.98 (−2.27, −1.69)
Australasia	160.1 (105.6, 235.9)	3.5 (2.3, 5.1)	90.2 (58.5, 132.9)	1.6 (1, 2.3)	−2.97 (−3.13, −2.81)
Caribbean	4762.2 (3334.4, 6510.7)	41.4 (29, 56.8)	2401.6 (1652.6, 3375.6)	20.9 (14.5, 29.3)	−2.25 (−2.4, −2.1)
Oceania	1430.6 (977.7, 1993.9)	53.6 (36.5, 75)	2200.7 (1470.2, 3055.5)	43.5 (28.9, 60.6)	−0.6 (−0.72, −0.47)
**Deaths**					
Global	275,290.1 (228,590.2, 320,314.9)	15.6 (13, 18.2)	70,658.7 (53,028.3, 89,634.3)	3.7 (2.8, 4.7)	−4.48 (−4.71, −4.24)
High SDI	579 (480, 705.6)	0.3 (0.3, 0.4)	23.3 (20.2, 27.6)	0 (0, 0)	−9.8 (−9.99, −9.61)
High–middle SDI	6961.6 (5884.3, 8017.8)	2.6 (2.2, 3)	368.5 (304, 443.8)	0.2 (0.1, 0.2)	−8.75 (−9.07, −8.42)
Middle SDI	49,506.8 (43,270.2, 55,308)	8.6 (7.5, 9.6)	5865.4 (4885.8, 7086.3)	1.1 (0.9, 1.3)	−6.34 (−6.49, −6.2)
Low–middle SDI	105,944.7 (83,580.5, 127,333.9)	21.7 (17.1, 26.1)	20,872.7 (16,366.2, 25,573)	3.7 (2.9, 4.6)	−5.47 (−5.63, −5.32)
Low SDI	112,153.1 (89,545.6, 134,928.6)	44.1 (35.2, 53.1)	43,484.4 (30,433.7, 57,872.5)	9.3 (6.5, 12.3)	−4.82 (−5.03, −4.61)
High-income North America	24.9 (24.1, 25.8)	0 (0, 0)	3.8 (3.4, 4.1)	0 (0, 0)	−6.06 (−6.65, −5.46)
Andean Latin America	2525.8 (2027.3, 3038.4)	16.8 (13.5, 20.3)	152.3 (114.3, 199.5)	0.9 (0.6, 1.1)	−9.49 (−9.77, −9.21)
Tropical Latin America	1183.4 (1044.8, 1344.5)	2.4 (2.1, 2.7)	107.8 (84.6, 134.4)	0.2 (0.2, 0.3)	−7.88 (−8.14, −7.62)
Central Latin America	1430.2 (1330.1, 1544)	2.2 (2, 2.4)	110 (88.4, 137.6)	0.2 (0.1, 0.2)	−8.39 (−8.99, −7.77)
Southern Latin America	152.7 (142.1, 164)	1 (1, 1.1)	14.3 (12.2, 16.7)	0.1 (0.1, 0.1)	−6.98 (−7.31, −6.66)
Western Europe	31.7 (30.4, 32.9)	0 (0, 0)	3.4 (3, 3.7)	0 (0, 0)	−7.06 (−7.2, −6.92)
Central Europe	155.3 (143.6, 168.2)	0.6 (0.5, 0.6)	12.5 (10.4, 14.9)	0.1 (0.1, 0.1)	−7.09 (−7.6, −6.57)
Eastern Europe	298.1 (283.1, 312.6)	0.6 (0.6, 0.6)	32.1 (29, 35.1)	0.1 (0.1, 0.1)	−5.64 (−6.85, −4.42)
High-income Asia Pacific	182.7 (140.8, 231.8)	0.5 (0.4, 0.7)	3.5 (3, 4.2)	0 (0, 0)	−10.73 (−11.08, −10.37)
Central Asia	1480.8 (1324.2, 1656.8)	5.5 (4.9, 6.2)	342.8 (268.7, 434.4)	1.2 (0.9, 1.5)	−5.26 (−5.87, −4.66)
South Asia	90,926.9 (71,217, 111,725.2)	20.5 (16.1, 25.2)	15,635.2 (12,501.8, 19,142.9)	3.3 (2.6, 4)	−5.77 (−5.86, −5.68)
Southeast Asia	31,494.3 (23,606.9, 38,080.6)	18.7 (14, 22.7)	4420.3 (3547.1, 5405.4)	2.6 (2.1, 3.2)	−6.15 (−6.32, −5.97)
East Asia	21444.1 (17,962.6, 25,339.3)	6.5 (5.4, 7.7)	399 (322.1, 500)	0.2 (0.1, 0.2)	−11.5 (−11.89, −11.12)
North Africa and Middle East	7639.7 (5988.6, 9742.6)	5.3 (4.1, 6.7)	1720.7 (1271.3, 2351.5)	1 (0.7, 1.3)	−5.29 (−5.59, −4.99)
Western Sub-Saharan Africa	33,466.9 (24,702.6, 42,222.3)	33.5 (24.7, 42.3)	19,641.2 (12,670.9, 30,082.7)	8.7 (5.6, 13.3)	−4.23 (−4.54, −3.91)
Eastern Sub-Saharan Africa	54,850.7 (42,975.5, 67,035.8)	54 (42.3, 66.1)	16,043.3 (11,464, 21,668.4)	8.8 (6.3, 11.9)	−5.64 (−5.76, −5.53)
Central Sub-Saharan Africa	22,110.2 (15,681, 29,008)	76.4 (54.1, 100.3)	8849.6 (5466.6, 13,932.7)	14.8 (9.2, 23.3)	−4.87 (−5.46, −4.28)
Southern Sub-Saharan Africa	4582.1 (3686.7, 6006.1)	21.6 (17.4, 28.4)	2600.6 (2039.6, 3318.5)	11.1 (8.7, 14.2)	−1.84 (−2.33, −1.35)
Australasia	1.4 (1.3, 1.6)	0 (0, 0)	0.2 (0.2, 0.3)	0 (0, 0)	−6.16 (−6.49, −5.83)
Caribbean	1036.4 (820.7, 1394.2)	8.9 (7, 11.9)	276.8 (180.3, 465.1)	2.5 (1.6, 4.1)	−3.66 (−4.01, −3.31)
Oceania	271.8 (155.3, 434.6)	9.8 (5.6, 15.7)	289.5 (166.1, 434.7)	5.4 (3.2, 8.2)	−1.61 (−1.99, −1.22)

Values in parentheses for case numbers and age-standardized rates indicate 95% uncertainty intervals (UIs) derived from GBD 2021 estimates. Values in parentheses for EAPC indicate 95% confidence intervals (CIs). Abbreviations: ASR, age-standardized rate; EAPC, estimated annual percentage change; SDI, socio-demographic index; UI, uncertainty interval; CI, confidence interval. Regional groupings follow the GBD 2021 classification. The list of countries/territories included in each regional category is provided in Table S2.

## Data Availability

Publicly available datasets were analyzed in this study. The data can be downloaded from the Global Health Data Exchange (GHDx) query tool (https://vizhub.healthdata.org/gbd-results/, accessed on 15 July 2024) and the official GBD 2021 platform (https://ghdx.healthdata.org/gbd-2021, accessed on 15 July 2024).

## Supplementary Materials

The following supporting information can be downloaded at: https://www.mdpi.com/article/10.3390/tropicalmed11050129/s1, Figure S1. Age-Period-Cohort (APC) Model Analysis of Childhood Tuberculosis Incidence and Mortality by Sex and Age Group; Figure S2. Health Inequality Analysis of Childhood Tuberculosis Incidence and Mortality in 1990 and 2021; Table S1 summarizes country-level trends in the childhood TB burden from 1990 to 2021; Table S2: Full list of countries and territories categorized by Socio-demographic Index (SDI) quintiles and GBD geographical regions.
