# Supplementary material for: Tailoring Prevention and Control Strategies for Childhood Tuberculosis: From a Global Analysis of Burden Trends and Inequalities Across Three Age Groups (1990–2021) to Prevention and Control Strategies
Source: Trop Med Infect Dis. 2026 May 9;11(5):129. doi: 10.3390/tropicalmed11050129 (PMC13211679; doi:10.3390/tropicalmed11050129)
Supplement: Supplementary file 1 [file tropicalmed-11-00129-s001.zip › Supplement_Figure.pdf]

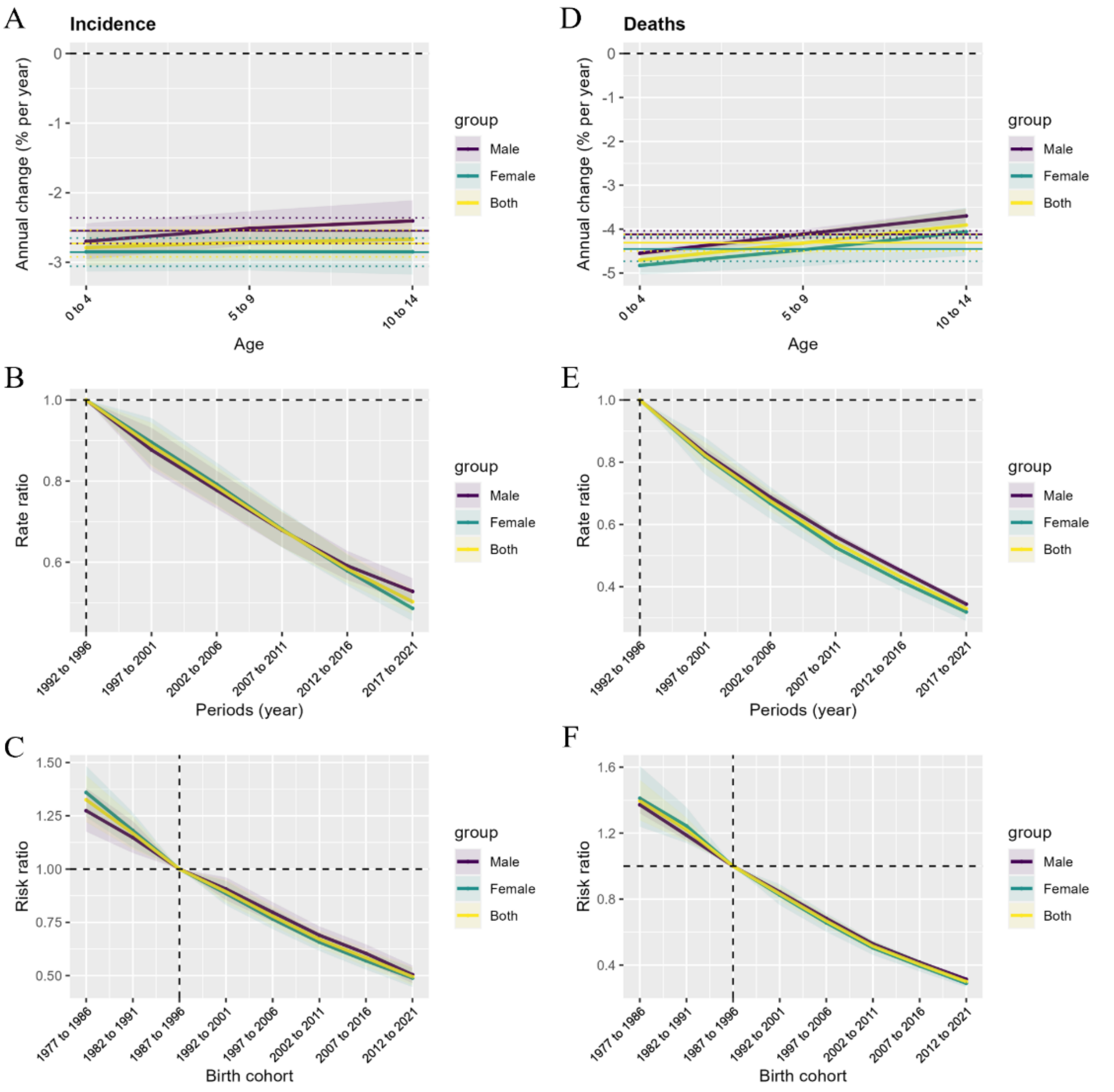

Figure S1. Age-Period-Cohort (APC) Model Analysis of Childhood Tuberculosis Incidence and Mortality by Sex and Age Group

A. Incidence Rate by Age Group

B. Incidence Rate by Period

C. Incidence Rate by Birth Cohort

D. Mortality Rate by Age Group

E. Mortality Rate by Period

F. Mortality Rate by Birth Cohort

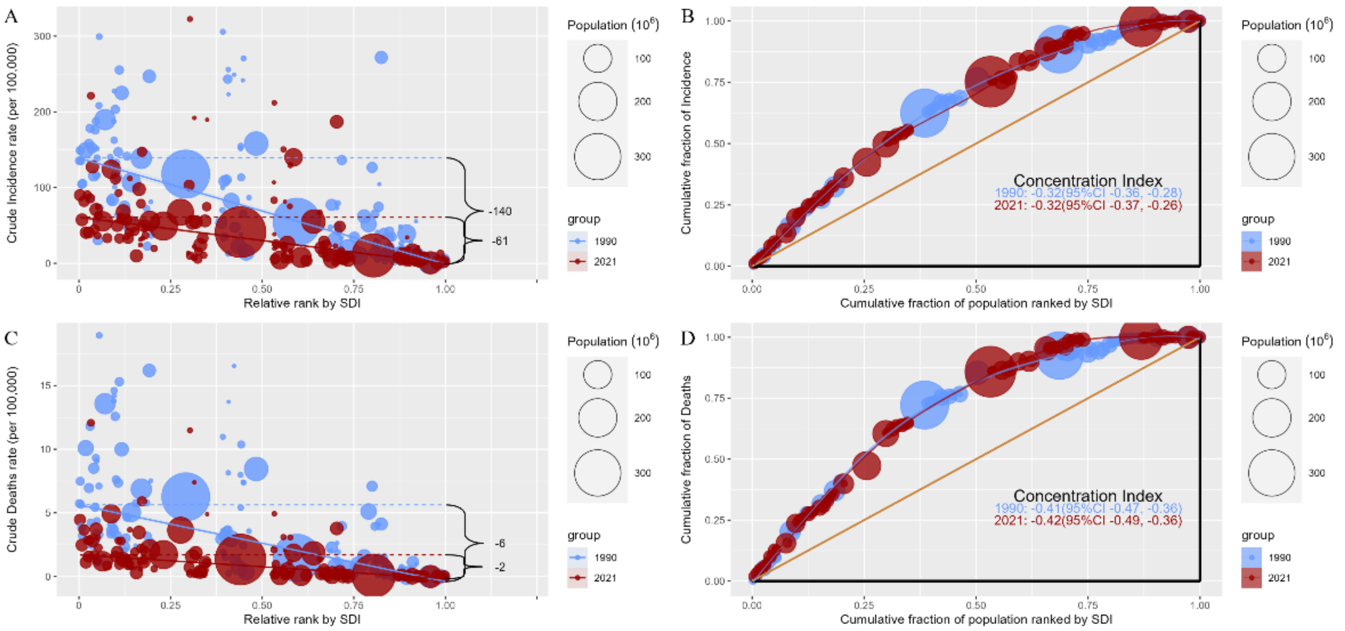

Figure S2. Health Inequality Analysis of Childhood Tuberculosis Incidence and Mortality in 1990 and 2021

A. Relative Risk of Incidence and SDI in 1990 and 2021

B. Cumulative Incidence Rate and Relative Risk by SDI in 1990 and 2021

C. Relative Risk of Mortality and SDI in 1990 and 2021

D. Cumulative Mortality Rate and Relative Risk by SDI in 1990 and 2021
