# Supplementary material for: Tailoring Prevention and Control Strategies for Childhood Tuberculosis: From a Global Analysis of Burden Trends and Inequalities Across Three Age Groups (1990–2021) to Prevention and Control Strategies
Source: Trop Med Infect Dis. 2026 May 9;11(5):129. doi: 10.3390/tropicalmed11050129 (PMC13211679; doi:10.3390/tropicalmed11050129)
Supplement: Supplementary file 1 [file tropicalmed-11-00129-s001.zip › Table_S1.pdf]

Table S1 summarizes country-level trends in the childhood TB burden from 1990 to 2021. In general, most countries showed declining age-standardized incidence rates (ASIR) and age-standardized mortality rates (ASMR) over time, although substantial geographic heterogeneity remained. In 2021, the highest childhood TB incidence burdens were observed in countries such as India, with 120,669.6 incident cases and an ASIR of 32.5 per 100,000, Nigeria, with 60,664.4 cases and an ASIR of 58.9 per 100,000, Pakistan, with 51,720.6 cases and an ASIR of 60.6 per 100,000, and the Philippines, with 40,912.7 cases and an ASIR of 119.8 per 100,000. By contrast, some countries exhibited particularly marked declines in incidence, including Turkey (EAPC in ASIR: -6.83, 95% CI: -7.02 to -6.63), Saudi Arabia (-6.25, 95% CI: -6.35 to -6.14), China (-5.69, 95% CI: -5.87 to -5.52), and the Republic of Korea (-5.81, 95% CI: -6.03 to -5.59). For mortality, high burdens in 2021 were still observed in Nigeria, with 11,585.0 deaths and an ASMR of 11.0 per 100,000, India, with 7,792.6 deaths and an ASMR of 2.3 per 100,000, Pakistan, with 6,627.7 deaths and an ASMR of 7.8 per 100,000, and the Democratic Republic of the Congo, with 5,347.0 deaths and an ASMR of 13.9 per 100,000. Mortality declined more rapidly than incidence in many settings, with particularly large decreases in Turkey (EAPC in ASMR: -13.34, 95% CI: -14.00 to -12.66), Serbia (-12.38, 95% CI: -12.97 to -11.78), China (-12.18, 95% CI: -12.60 to -11.76), and Saudi Arabia (-11.23, 95% CI: -11.31 to -11.15). However, a few countries showed stable or increasing trends, such as the Philippines for incidence (EAPC: 4.43, 95% CI: 2.91 to 5.97), the United States for incidence (EAPC: 1.52, 95% CI: 0.88 to 2.17), and Zimbabwe for mortality (EAPC: 1.12, 95% CI: 0.61 to 1.62).

Table S1: Country trends in childhood tuberculosis burden: incidence, and mortality (1990–2021).

Location	1990		2021		EAPC_95%CI
	Number (95%UI)	ASR (95%UI)	Number (95%UI)	ASR (95%UI)	
**Incidence**					
Afghanistan	7536.9 (5311.3, 10387.1)	172.5 (120.3, 239.5)	7489.2 (4965.2, 10632.7)	53.4 (35, 76.1)	-3.04 (-3.72, -2.34)
Albania	104.7 (70, 149.8)	9.3 (6.2, 13.4)	15.3 (10.1, 22.2)	3.5 (2.3, 5)	-3.44 (-3.55, -3.33)
Algeria	6874.2 (4675.7, 9739.6)	64.4 (43.8, 91.2)	2107 (1359.9, 3053)	16 (10.3, 23.1)	-4.39 (-4.42, -4.37)
American Samoa	1.5 (1, 2.2)	8.1 (5.3, 11.7)	0.5 (0.3, 0.7)	3.5 (2.3, 5.1)	-3.11 (-3.52, -2.71)
Andorra	0.5 (0.3, 0.7)	5.6 (3.7, 8.1)	0.2 (0.1, 0.3)	2 (1.3, 2.9)	-3.24 (-3.5, -2.99)
Angola	12636.2 (9232, 16910.7)	253.9 (182.7, 343.8)	18541.2 (13109.1, 25434.6)	119.9 (84.3, 165.2)	-2.51 (-2.82, -2.2)
Antigua and Barbuda	3.1 (2.1, 4.6)	17.3 (11.4, 25.4)	1.3 (0.8, 1.9)	7.6 (5, 11)	-2.56 (-2.66, -2.46)
Argentina	1067.3 (686.6, 1571.7)	10.5 (6.8, 15.4)	598 (382.9, 874.1)	5.7 (3.7, 8.3)	-2.43 (-2.63, -2.24)
Armenia	139.2 (93, 200.7)	13.3 (8.9, 19.3)	28.2 (18.7, 41)	4.8 (3.2, 6.9)	-2.87 (-3.34, -2.4)
Australia	114.9 (75.2, 170.7)	3.1 (2, 4.5)	62 (40.6, 90.8)	1.3 (0.9, 1.9)	-3.01 (-3.14, -2.89)
Austria	68.7 (44.7, 102.4)	5.1 (3.3, 7.5)	30.3 (19.1, 46.2)	2.3 (1.5, 3.5)	-2.47 (-2.61, -2.34)
Azerbaijan	782.8 (535.5, 1096.5)	32.1 (21.8, 45.1)	310.1 (207.1, 436.4)	13.1 (8.8, 18.4)	-3.34 (-3.76, -2.91)
Bahamas	10.4 (6.8, 15.2)	12.9 (8.5, 18.7)	4.1 (2.6, 6)	5 (3.3, 7.2)	-3.25 (-3.4, -3.09)
Bahrain	19.5 (13.3, 27.7)	12 (8.1, 17.2)	15 (9.7, 21.9)	5 (3.3, 7.3)	-3.21 (-3.49, -2.92)
Bangladesh	83208.7 (56043.5, 116692.4)	170.6 (114, 240.7)	19098.1 (12607.6, 27374.4)	40.9 (27.2, 58.4)	-4.5 (-4.58, -4.42)
Barbados	3.4 (2.2, 5.1)	5.5 (3.6, 8.1)	1.1 (0.7, 1.7)	2.4 (1.5, 3.5)	-2.6 (-2.72, -2.48)
Belarus	341.1 (217.1, 515.9)	14.2 (9, 21.4)	45.1 (29.1, 66.6)	2.9 (1.9, 4.2)	-6.11 (-6.59, -5.63)
Belgium	81.3 (51.6, 120)	4.5 (2.9, 6.6)	53.5 (33.7, 80.5)	2.8 (1.8, 4.2)	-1.53 (-1.62, -1.45)

Location	1990		2021		EAPC_95%CI
	Number (95%UI)	ASR (95%UI)	Number (95%UI)	ASR (95%UI)	
Belize	24.3 (16.4, 34.7)	29.9 (20.1, 42.6)	10.5 (6.6, 15.2)	8.3 (5.3, 12)	-4.85 (-5.11, -4.6)
Benin	3009.5 (2306.5, 3763.6)	115.9 (88.1, 145.8)	2625.1 (1847.2, 3636.6)	41.8 (29.2, 58.2)	-3.34 (-3.59, -3.09)
Bermuda	2.1 (1.4, 3.1)	17.6 (11.6, 25.8)	0.5 (0.3, 0.8)	6.1 (4, 9)	-3.35 (-3.51, -3.19)
Bhutan	148.1 (98.8, 214.8)	56.3 (37.4, 81.8)	38.2 (25, 55.8)	20.2 (13.3, 29.4)	-3.48 (-3.66, -3.31)
Bolivia (Plurinational State of)	2703.5 (1854.5, 3834.6)	100.4 (68.4, 143.1)	726.2 (500.4, 1018.8)	20.8 (14.4, 29.2)	-5.27 (-5.38, -5.16)
Bosnia and Herzegovina	116 (76.5, 171.3)	10.6 (7, 15.6)	19.9 (13, 29.4)	4 (2.6, 5.9)	-3.21 (-3.39, -3.04)
Botswana	811.2 (547.4, 1159.6)	136.9 (92.1, 196)	902.3 (618.6, 1255.9)	129.7 (89.3, 180.1)	-1.5 (-2.37, -0.63)
Brazil	8075 (5406, 11451.8)	15.8 (10.7, 22.2)	2887.6 (1889.1, 4166.1)	6 (3.9, 8.6)	-4.65 (-5.38, -3.92)
Brunei Darussalam	9.4 (6.1, 13.6)	10.4 (6.8, 15.2)	4 (2.5, 6)	4.1 (2.6, 6.2)	-3.17 (-3.34, -3.01)
Bulgaria	139.6 (88.5, 209.6)	8 (5.1, 11.9)	59.6 (38.8, 86.1)	6 (3.9, 8.6)	0.14 (-0.38, 0.66)
Burkina Faso	8429.9 (6099.7, 11210.6)	168.1 (120.4, 225.2)	6445.1 (4640.5, 8715.4)	58.7 (41.8, 80.1)	-3.22 (-3.35, -3.09)
Burundi	6000.4 (4177.9, 8310.3)	219.1 (150.4, 306.6)	5374 (3728.8, 7496.3)	90.8 (62.6, 127)	-3.37 (-3.63, -3.1)
Cabo Verde	160.6 (116, 216)	98.8 (70.8, 133.6)	35.5 (24.7, 49.6)	25.6 (18, 35.4)	-4.52 (-4.63, -4.41)
Cambodia	10135.3 (7536.8, 12760.1)	217.5 (160.9, 274.3)	3394.8 (2322.2, 4859.3)	66.3 (45.4, 94.7)	-3.84 (-4.17, -3.51)
Cameroon	5404.3 (3889.9, 7187.2)	103.3 (73.4, 138.7)	7901.3 (5436.1, 11005.9)	58.2 (39.9, 81.2)	-2.28 (-2.87, -1.69)
Canada	79.8 (51.1, 118.2)	1.4 (0.9, 2.1)	85.5 (55.4, 125.5)	1.4 (0.9, 2)	-0.1 (-0.23, 0.02)
Central African Republic	4915.4 (3491.7, 6551.7)	381.9 (268, 514.1)	6039.8 (4140.2, 8417.6)	260.5 (177.7, 364.4)	-1.31 (-1.38, -1.23)
Chad	6624 (4895.3, 8796.9)	207.6 (151, 278.8)	9021.3 (6434.8, 12294.1)	96.2 (67.6, 132.4)	-2.31 (-2.36, -2.25)
Chile	459.5 (305.3, 658.1)	11.6 (7.7, 16.6)	99.1 (62.7, 146.3)	2.7 (1.7, 3.9)	-5.37 (-5.79, -4.94)
China	169980.1 (115539.6, 235542.6)	53.2 (36.2, 73.7)	23905.6 (16632.4, 33496.7)	9.5 (6.7, 13.2)	-5.69 (-5.87, -5.52)
Colombia	1552.2 (1058.1, 2217.4)	13.2 (9, 19)	351.5 (241.6, 496.7)	3.3 (2.3, 4.7)	-5.01 (-5.23, -4.78)
Comoros	275.9 (196.3, 379.6)	125.3 (88.4, 173.8)	107.9 (75.6, 147.1)	45.4 (31.9, 61.8)	-3.4 (-3.52, -3.28)
Congo	2115.3 (1499.1, 2917.3)	196.5 (138.6, 272.2)	1736.2 (1137.9, 2482.9)	91.5 (60.4, 130.3)	-2.47 (-2.77, -2.17)
Cook Islands	0.6 (0.4, 0.8)	8.4 (5.5, 12.3)	0.1 (0.1, 0.2)	3.2 (2.1, 4.7)	-3.55 (-3.68, -3.42)
Costa Rica	60.8 (40.8, 86.7)	5.4 (3.6, 7.7)	15.8 (10.2, 23.5)	1.6 (1, 2.3)	-4.94 (-5.31, -4.56)
Coted'Ivoire	10357 (7472.2, 13788.2)	170.6 (121.6, 229.2)	7444 (5244.8, 10233.1)	63.3 (44.3, 87.4)	-2.94 (-3.02, -2.86)
Croatia	77.7 (49.7, 115)	7.8 (5.1, 11.5)	12.2 (7.9, 18.2)	2 (1.3, 3)	-4.72 (-4.87, -4.56)
Cuba	99.6 (66.5, 143.1)	3.9 (2.6, 5.7)	24.4 (16.3, 35.3)	1.4 (1, 2)	-3.49 (-3.74, -3.24)
Cyprus	6.5 (4.3, 9.5)	3.3 (2.2, 4.8)	2.9 (1.9, 4.4)	1.3 (0.9, 2)	-2.94 (-3.07, -2.8)
Czechia	80.5 (50.9, 121.9)	3.6 (2.3, 5.4)	20.7 (13.1, 31.5)	1.2 (0.8, 1.8)	-3.91 (-4.17, -3.64)
Democratic People's Republic of Korea	7702.5 (5386.4, 10732.2)	126.5 (87.5, 177.9)	3336.2 (2323.3, 4727.6)	70.4 (49.5, 99)	-1.93 (-2.31, -1.54)

Location	1990		2021		EAPC_95%CI
	Number (95%UI)	ASR (95%UI)	Number (95%UI)	ASR (95%UI)	
Democratic Republic of the Congo	52358.6 (37237.4, 71567.6)	280.6 (196.8, 387.5)	57983.8 (41031.4, 79329.8)	151.8 (107.2, 208)	-1.93 (-2.2, -1.66)
Denmark	38.9 (24.8, 58.7)	4.4 (2.8, 6.6)	19.1 (11.9, 28.8)	2 (1.3, 3)	-3.71 (-4.12, -3.29)
Djibouti	246.6 (183.9, 318.9)	137.8 (102.5, 178.7)	202.8 (136.4, 287.1)	49 (32.9, 69.4)	-3.4 (-3.79, -3.01)
Dominica	3.3 (2.2, 4.6)	13.1 (8.9, 18.4)	0.9 (0.6, 1.3)	6.3 (4.3, 9)	-2.2 (-2.4, -2)
Dominican Republic	1462.7 (1000.7, 2024.7)	53.9 (36.8, 74.9)	457.9 (313.7, 654.5)	15.5 (10.6, 22.2)	-4.18 (-4.37, -4)
Ecuador	1863.8 (1264.9, 2652.7)	48.1 (32.7, 68.5)	520.3 (345.2, 746.2)	10.2 (6.8, 14.5)	-6.03 (-6.32, -5.74)
Egypt	3914 (2737.4, 5222.2)	17.6 (12.2, 23.6)	1276 (871, 1808.4)	3.5 (2.4, 4.9)	-4.48 (-4.76, -4.21)
El Salvador	804.5 (552.8, 1107.9)	37.1 (25.5, 51.2)	144 (94.7, 210.9)	7.9 (5.2, 11.6)	-5 (-5.27, -4.73)
Equatorial Guinea	534.6 (379.6, 719.3)	258.6 (180.9, 351.9)	426.8 (286.2, 601.4)	74.4 (50.4, 104.2)	-4.62 (-4.88, -4.37)
Eritrea	4651.1 (3327.1, 6267.1)	279.1 (197.6, 378.9)	3169.1 (2255.1, 4280.1)	123.7 (87.7, 167.6)	-2.47 (-2.58, -2.37)
Estonia	38.3 (24.2, 57.9)	11 (6.9, 16.6)	7.8 (5, 11.9)	3.6 (2.3, 5.4)	-3.98 (-4.18, -3.78)
Eswatini	988.7 (687.1, 1386.7)	253.5 (175.2, 357.3)	778.2 (461.5, 1172.8)	188.7 (112.5, 283.6)	-0.73 (-0.84, -0.61)
Ethiopia	49006.7 (34664.8, 67888.3)	192.3 (134.5, 268.5)	28129.1 (19592.3, 38848.9)	62.8 (43.6, 86.9)	-3.88 (-4, -3.75)
Fiji	76.5 (49.7, 111.5)	27.1 (17.6, 39.4)	43.4 (29.1, 62.6)	15.8 (10.6, 22.8)	-2.13 (-2.36, -1.9)
Finland	36.2 (23.3, 54.1)	3.7 (2.4, 5.6)	15.5 (9.5, 23.9)	1.8 (1.1, 2.8)	-2.53 (-2.64, -2.42)
France	709.3 (454.6, 1040.8)	6 (3.9, 8.8)	248.1 (156.1, 373.3)	2.1 (1.3, 3.2)	-3.71 (-4.05, -3.36)
Gabon	468.1 (332.7, 640.8)	111.3 (78.6, 153.4)	477.1 (319.4, 674.6)	75.3 (50.7, 106.1)	-1.38 (-1.93, -0.83)
Gambia	1070.4 (798.7, 1424.3)	216.5 (159.7, 290.9)	825.9 (582.5, 1132.2)	82.2 (57.8, 112.9)	-3.14 (-3.24, -3.05)
Georgia	248.8 (167, 356.9)	18.2 (12.2, 26.1)	82.9 (54.9, 118.1)	11.2 (7.5, 16)	-0.29 (-1.06, 0.48)
Germany	520.6 (335, 764.3)	4 (2.6, 5.9)	234.4 (152.8, 346)	2 (1.3, 2.9)	-2.41 (-2.7, -2.12)
Ghana	11163.8 (7964.5, 15105.8)	159.1 (112.5, 216.6)	7291.6 (4918.2, 10190.6)	56.4 (37.9, 78.9)	-3.08 (-3.19, -2.98)
Greece	57.9 (34.9, 88.9)	2.8 (1.7, 4.3)	19.7 (12, 30.5)	1.4 (0.9, 2.2)	-2.12 (-2.22, -2.02)
Greenland	2.6 (1.7, 3.8)	18.2 (12, 26.7)	1.7 (1, 2.5)	14.2 (8.9, 21.2)	0.11 (-0.23, 0.46)
Grenada	3.7 (2.4, 5.4)	11 (7.2, 16.3)	1 (0.7, 1.5)	4.8 (3.1, 7)	-2.65 (-2.77, -2.53)
Guam	7.6 (5.1, 11.1)	18.7 (12.3, 27.2)	4.9 (3.2, 7.1)	13.2 (8.7, 19.1)	-1.58 (-1.75, -1.42)
Guatemala	884.9 (635, 1184.1)	21.6 (15.5, 29)	240.9 (164.7, 337.8)	4.9 (3.4, 6.8)	-4.49 (-4.74, -4.24)
Guinea	4151.5 (3044.9, 5509.6)	138 (99.7, 185.4)	3455.8 (2408.5, 4740.8)	56 (38.8, 77.3)	-2.71 (-2.98, -2.43)
Guinea-Bissau	1108.7 (797.3, 1493.8)	220.2 (157.1, 298.3)	707.3 (457, 1042.4)	77.8 (50, 115)	-3.11 (-3.18, -3.05)
Guyana	111.9 (75.7, 156.9)	37.8 (25.4, 53.3)	25.8 (17.6, 36.3)	12.1 (8.2, 17)	-3.78 (-3.91, -3.66)
Haiti	2726.8 (1893.2, 3745.5)	99.1 (68.3, 136.8)	1756.7 (1186.8, 2480.1)	40.2 (27.1, 56.9)	-2.87 (-3, -2.74)
Honduras	691.4 (469.5, 956.1)	31.4 (21.3, 43.5)	250.8 (166.6, 358.3)	7.6 (5.1, 10.8)	-4.88 (-5, -4.77)

Location	1990		2021		EAPC_95%CI
	Number (95%UI)	ASR (95%UI)	Number (95%UI)	ASR (95%UI)	
Hungary	133.2 (84.7, 199.5)	6.2 (4, 9.2)	19.1 (12.4, 28.1)	1.4 (0.9, 2)	-5.69 (-6.05, -5.32)
Iceland	2.2 (1.4, 3.4)	3.5 (2.2, 5.3)	1.4 (0.8, 2.2)	2 (1.2, 3.1)	-1.69 (-1.78, -1.59)
India	327207.2 (201563.4, 490433.3)	100.7 (61.9, 151.2)	120669.6 (80338.4, 170113)	32.5 (21.8, 45.5)	-4.29 (-4.5, -4.07)
Indonesia	95427.3 (64820.9, 132990.5)	141.3 (96.4, 196.5)	32829.8 (22864.1, 45207.3)	48.9 (34.3, 67)	-3.55 (-3.66, -3.45)
Iran (Islamic Republic of)	3645.8 (2522.7, 5102.2)	14.4 (10, 20.2)	1125.5 (751.1, 1615.3)	5.5 (3.7, 7.9)	-2.71 (-3.08, -2.35)
Iraq	3158.1 (2148.4, 4491.7)	38.7 (26.1, 55.2)	1095.8 (720.5, 1554.3)	8.1 (5.4, 11.4)	-5.12 (-5.47, -4.75)
Ireland	49.2 (30.6, 75.6)	4.9 (3.1, 7.5)	20.4 (12.4, 31.4)	2 (1.2, 3.1)	-3.27 (-3.42, -3.11)
Israel	48.2 (30.3, 72.6)	3.1 (2, 4.7)	32.2 (20.2, 48.8)	1.2 (0.8, 1.9)	-3.63 (-3.85, -3.42)
Italy	442.8 (277.8, 674.7)	4.7 (3, 7.1)	193.5 (117.6, 299.4)	2.5 (1.6, 3.8)	-2.14 (-2.24, -2.04)
Jamaica	34.6 (22.2, 51.5)	4.2 (2.7, 6.2)	10.3 (6.7, 15.2)	1.8 (1.2, 2.6)	-2.78 (-2.89, -2.66)
Japan	370.4 (215.9, 593.7)	1.6 (0.9, 2.5)	70.1 (40.4, 112.2)	0.5 (0.3, 0.7)	-4.37 (-4.72, -4.02)
Jordan	106.4 (69.5, 153.4)	6.5 (4.3, 9.5)	79.6 (46.6, 123.5)	2.1 (1.3, 3.3)	-3.95 (-4.19, -3.71)
Kazakhstan	1857.7 (1219, 2733.6)	35.7 (23.4, 52.7)	482.7 (328.3, 682.9)	8.9 (6.1, 12.6)	-4.72 (-5.74, -3.69)
Kenya	12071.4 (8718.2, 16232.6)	102.7 (73.7, 138.8)	8704.7 (5477.8, 12819)	46.8 (29.7, 68.4)	-3.02 (-3.44, -2.6)
Kiribati	55 (36.9, 77.3)	190 (126.3, 267.6)	54.5 (36, 76)	129 (85.4, 180)	-1.35 (-1.44, -1.26)
Kuwait	102 (66.3, 147)	18.6 (12.1, 26.9)	38.3 (24.5, 56.3)	4.5 (2.9, 6.5)	-5.06 (-5.23, -4.89)
Kyrgyzstan	441 (304, 615.1)	25.9 (17.7, 36.3)	395.4 (266.6, 557.2)	17.5 (11.8, 24.6)	-0.3 (-0.67, 0.08)
Lao People's Democratic Republic	2593.8 (1781.7, 3718.6)	140.8 (95.8, 203)	752.1 (508.2, 1074.1)	32.7 (22, 46.8)	-4.95 (-5.13, -4.77)
Latvia	90.4 (57.3, 134.8)	15.9 (10.1, 23.8)	16.2 (10.4, 24.2)	5.4 (3.5, 8)	-3.02 (-3.47, -2.57)
Lebanon	145.6 (97.6, 206.7)	13.9 (9.3, 19.8)	42.7 (27.7, 61.9)	3.3 (2.2, 4.8)	-4.76 (-4.89, -4.64)
Lesotho	1983.4 (1347.8, 2743.8)	289.9 (196.6, 401.5)	1706.5 (993.9, 2578.8)	270.5 (158.8, 406.4)	-0.02 (-0.26, 0.23)
Liberia	2580.1 (1893.5, 3443.5)	213.3 (154.4, 287.7)	1027 (717.5, 1415.8)	46.9 (32.8, 64.7)	-5.51 (-5.83, -5.19)
Libya	430.4 (293, 607.7)	23.7 (16.1, 33.5)	164.6 (106.4, 239.9)	11 (7.2, 15.8)	-2.31 (-2.58, -2.05)
Lithuania	131.1 (87, 192.8)	15.8 (10.5, 23.2)	32.4 (21.1, 47.4)	7.9 (5.1, 11.5)	-1.38 (-1.87, -0.88)
Luxembourg	5 (3.2, 7.6)	7.6 (4.9, 11.4)	4.3 (2.6, 6.7)	4.2 (2.6, 6.6)	-1.78 (-1.85, -1.71)
Madagascar	10344 (7331.7, 13892.2)	182 (127.6, 246.5)	7358.4 (5341.7, 10060.5)	62.9 (45.6, 85.9)	-3.01 (-3.12, -2.9)
Malawi	9305.5 (6528.8, 12641.7)	194.8 (135.7, 266.1)	9032.9 (5636.2, 13318.3)	111.6 (70.1, 163.9)	-2.85 (-3.26, -2.45)
Malaysia	1903 (1280.4, 2707)	29.1 (19.5, 41.5)	869.6 (575.8, 1268.5)	11.3 (7.5, 16.4)	-3.25 (-3.4, -3.11)
Maldives	60.7 (40.1, 87.3)	59.3 (38.8, 85.6)	13.3 (8.9, 19)	13.3 (8.9, 18.9)	-5.26 (-5.47, -5.04)
Mali	7389.5 (5453.2, 9890.7)	164.7 (119.8, 222.8)	6343.4 (4448.3, 8804.7)	52.4 (36.3, 73.3)	-3.62 (-3.66, -3.58)
Malta	16.5 (10.5, 25.1)	18.8 (12, 28.5)	7 (4.3, 10.9)	10.9 (6.8, 17)	-1.59 (-1.67, -1.5)

Location	1990		2021		EAPC_95%CI
	Number (95%UI)	ASR (95%UI)	Number (95%UI)	ASR (95%UI)	
Marshall Islands	36.3 (23.7, 51.4)	166.9 (108.9, 235.8)	22.7 (14.9, 32.3)	127.5 (84.3, 181.3)	-0.88 (-1.15, -0.62)
Mauritania	960.3 (704.1, 1274.7)	98.2 (71.3, 131.5)	483.6 (347.2, 657.3)	26 (18.6, 35.4)	-4 (-4.09, -3.9)
Mauritius	22.5 (14.8, 33.7)	6.7 (4.5, 10)	9 (5.8, 13.3)	4.3 (2.8, 6.3)	-1.44 (-1.53, -1.36)
Mexico	2855.3 (1955.6, 3947.3)	8.5 (5.8, 11.7)	1105 (695.7, 1674.2)	3.4 (2.1, 5.1)	-4.19 (-4.79, -3.58)
Micronesia (Federated States of)	23.6 (15.4, 33.5)	51.5 (33.5, 72.8)	8.3 (5.5, 11.9)	26.3 (17.7, 37.6)	-2.16 (-2.24, -2.07)
Monaco	0.1 (0.1, 0.2)	3 (1.9, 4.5)	0.1 (0, 0.1)	1.1 (0.7, 1.7)	-3.23 (-3.42, -3.04)
Mongolia	760.5 (525.1, 1036.5)	83.2 (57.1, 113.9)	230.9 (157.4, 321.8)	21.5 (14.6, 30)	-4.31 (-4.55, -4.07)
Montenegro	7.4 (4.7, 11)	4.6 (3, 6.8)	2.1 (1.4, 3.1)	1.9 (1.2, 2.8)	-3.33 (-3.55, -3.1)
Morocco	9098 (6228, 12981.8)	92.9 (63.4, 132.8)	2407.1 (1584.4, 3496.9)	24.4 (16.1, 35.3)	-4.3 (-4.45, -4.15)
Mozambique	15126.1 (10840.4, 20643.1)	233.9 (166.1, 321.9)	17944.6 (10999.2, 26286.7)	125.5 (76.3, 184.7)	-1.89 (-2.05, -1.73)
Myanmar	28607.5 (19341.3, 39713.6)	193 (130.7, 267.8)	7385.6 (4909.7, 10548.7)	47.2 (31.5, 67.2)	-4.71 (-4.91, -4.52)
Namibia	1626.3 (1141.8, 2236.2)	266.4 (185.8, 367.9)	1162.2 (758.4, 1690)	141.1 (92.6, 204.6)	-1.8 (-1.97, -1.63)
Nauru	3 (2, 4.3)	73.6 (48.9, 105.5)	2.3 (1.5, 3.2)	56.9 (38.1, 80.9)	-0.8 (-1.21, -0.38)
Nepal	9336.6 (7196, 11734.8)	109.4 (83.7, 138.4)	2586.9 (1764, 3636.7)	27.9 (19.1, 39.1)	-4.67 (-4.81, -4.54)
Netherlands	147.9 (93.1, 220.6)	5.4 (3.4, 8)	42.9 (26.9, 64.8)	1.6 (1, 2.4)	-4.82 (-5.31, -4.32)
New Zealand	45.2 (29.2, 67.2)	5.6 (3.6, 8.3)	28.2 (17.7, 43.1)	2.9 (1.8, 4.4)	-2.81 (-3.1, -2.53)
Nicaragua	623 (428.2, 863.8)	34.2 (23.4, 47.5)	127.8 (85.4, 181.8)	6.5 (4.3, 9.2)	-5.74 (-5.91, -5.56)
Niger	9878.5 (7106.7, 13197.7)	226.4 (160.7, 305.8)	9859.6 (7048.4, 13305.4)	74 (52.4, 100.6)	-3.77 (-3.88, -3.66)
Nigeria	71464.4 (52853.2, 94209.8)	170.5 (124.6, 226.8)	60664.4 (43087.3, 82478.6)	58.9 (41.6, 80.3)	-3.46 (-3.85, -3.06)
Niue	0.1 (0.1, 0.2)	18.5 (12.2, 26.9)	0.1 (0, 0.1)	12.7 (8.6, 18.2)	-1.39 (-1.52, -1.26)
North Macedonia	50.4 (34.6, 73)	9.7 (6.7, 14)	7.8 (5.2, 11.4)	2.4 (1.6, 3.5)	-4.6 (-4.79, -4.42)
Northern Mariana Islands	4.8 (3.2, 7)	39.8 (26.3, 57.9)	3 (2, 4.4)	26.1 (17.2, 37.5)	-1.19 (-1.31, -1.07)
Norway	25.1 (15.9, 37.3)	3.1 (2, 4.6)	15.9 (9.4, 25.7)	1.7 (1, 2.7)	-2.13 (-2.21, -2.05)
Oman	99.6 (67.1, 141.4)	11.9 (7.9, 17)	38.9 (25.1, 56.6)	3.2 (2.1, 4.7)	-4.05 (-4.29, -3.81)
Pakistan	67308.8 (46762.1, 93015.4)	136.1 (94, 188.8)	51720.6 (35355, 72453)	60.6 (41.4, 84.8)	-2.64 (-2.8, -2.48)
Palau	0.8 (0.5, 1.2)	17.5 (11.5, 25.6)	0.4 (0.3, 0.5)	11.3 (7.7, 16)	-1.37 (-1.45, -1.29)
Palestine	109.4 (75.4, 156)	11.3 (7.6, 16.2)	64.1 (42.1, 93.5)	3.4 (2.3, 5)	-3.68 (-3.78, -3.59)
Panama	254.3 (170.1, 361.6)	30.5 (20.4, 43.3)	94.8 (63.8, 135.8)	8.2 (5.6, 11.7)	-3.98 (-4.4, -3.56)
Papua New Guinea	1028.4 (698.4, 1440.7)	60.7 (40.9, 85.4)	1850.8 (1234.5, 2572.6)	47.5 (31.5, 66.3)	-0.68 (-0.82, -0.54)
Paraguay	224.7 (152.1, 316.8)	13.5 (9.1, 19.1)	129.9 (87.4, 184.3)	6.4 (4.4, 9.1)	-2.58 (-2.72, -2.44)
Peru	8093.4 (5424.7, 11717.2)	97.4 (65.2, 141)	1918.7 (1269.9, 2747.2)	20.1 (13.3, 28.7)	-5.61 (-6.11, -5.12)

Location	1990		2021		EAPC_95%CI
	Number (95%UI)	ASR (95%UI)	Number (95%UI)	ASR (95%UI)	
Philippines	10958.6 (7695.5, 15007.2)	43.3 (30.3, 59.4)	40912.7 (27752, 57392.5)	119.8 (81.6, 167.7)	4.43 (2.91, 5.97)
Poland	450.6 (275.7, 701.3)	4.7 (2.9, 7.3)	119.6 (75.9, 181.3)	2 (1.3, 3.1)	-2.14 (-2.69, -1.58)
Portugal	294.8 (188.1, 440.5)	13.2 (8.6, 19.5)	44.4 (27.7, 67.3)	3.2 (2, 4.8)	-5.18 (-5.41, -4.95)
Puerto Rico	38.5 (25, 56.9)	3.8 (2.5, 5.6)	5.1 (3.1, 8)	1.2 (0.7, 1.8)	-4.03 (-4.15, -3.92)
Qatar	47.2 (31.9, 67)	38 (25.4, 54.4)	44.5 (28.8, 66)	9.1 (5.9, 13.6)	-4.8 (-4.91, -4.69)
Republic of Korea	1358.1 (1000.5, 1786.9)	11.8 (8.7, 15.4)	104.8 (66.4, 156)	1.7 (1.1, 2.5)	-5.81 (-6.03, -5.59)
Republic of Moldova	252.5 (162.9, 376.7)	20.4 (13.2, 30.5)	64.7 (42.3, 94.3)	12.1 (7.9, 17.5)	-0.78 (-1.07, -0.48)
Romania	1835.7 (1213.9, 2653.5)	32.8 (21.8, 47.2)	342.1 (226.9, 487.4)	11.2 (7.5, 15.8)	-3.51 (-3.65, -3.38)
Russian Federation	10052.1 (6460.5, 15008.2)	28.9 (18.6, 43)	2702.2 (1671.8, 4043.5)	10 (6.2, 14.9)	-1.58 (-2.15, -1.01)
Rwanda	5077 (3492.4, 7100.9)	145.7 (99.2, 205.4)	2176.3 (1464.1, 3046)	43.7 (29.4, 61.1)	-3.75 (-3.92, -3.58)
Saint Kitts and Nevis	1.4 (1, 2)	10 (6.9, 14.1)	0.3 (0.2, 0.4)	3.1 (2.1, 4.4)	-3.55 (-3.73, -3.37)
Saint Lucia	6.1 (4.1, 9)	11.9 (7.9, 17.3)	1.2 (0.7, 1.7)	3.9 (2.5, 5.7)	-3.67 (-3.87, -3.46)
Saint Vincent and the Grenadines	6.6 (4.3, 9.6)	16 (10.5, 23.1)	1.5 (0.9, 2.2)	5.9 (3.8, 8.6)	-3.53 (-3.67, -3.39)
Samoa	17.2 (11.5, 25.1)	24.1 (16.1, 35.1)	11.4 (7.6, 16.4)	14.3 (9.6, 20.7)	-1.74 (-1.79, -1.69)
San Marino	0.1 (0, 0.1)	1.7 (1.1, 2.4)	0 (0, 0)	0.6 (0.4, 0.9)	-3.37 (-3.51, -3.23)
Sao Tome and Principe	46.7 (33.9, 62.3)	81.4 (58.9, 108.9)	14.4 (10.4, 19.7)	19.1 (13.9, 26)	-4.59 (-4.69, -4.5)
Saudi Arabia	3678.6 (2518.6, 5189.2)	56.8 (38.7, 80.2)	657 (413.9, 968.5)	8.6 (5.4, 12.6)	-6.25 (-6.35, -6.14)
Senegal	4613.3 (3377.1, 6200.3)	119.2 (86.3, 161.8)	2471.3 (1734.2, 3424.6)	38.6 (27, 53.5)	-3.77 (-3.95, -3.59)
Serbia	152.3 (101.4, 227)	7 (4.7, 10.4)	37.5 (24.1, 55.2)	2.7 (1.8, 3.9)	-3.31 (-3.49, -3.14)
Seychelles	2.5 (1.6, 3.6)	10.3 (6.9, 15.1)	1.4 (1, 2)	6 (4.1, 8.7)	-1.69 (-1.78, -1.59)
Sierra Leone	4591.4 (3402.7, 6094.1)	230.1 (167.9, 308.9)	3187.3 (2279.9, 4291.4)	86.7 (61.7, 117.3)	-3.17 (-3.51, -2.82)
Singapore	35.8 (23, 52.9)	5.5 (3.6, 8)	12 (7.4, 18.3)	1.5 (0.9, 2.3)	-4.54 (-4.68, -4.4)
Slovakia	34.1 (22.3, 50)	2.6 (1.7, 3.8)	6.9 (4.6, 9.9)	0.8 (0.5, 1.2)	-3.81 (-3.89, -3.73)
Slovenia	18.1 (11.4, 27.3)	4.4 (2.8, 6.5)	4.7 (2.9, 7.1)	1.5 (0.9, 2.3)	-3.66 (-3.75, -3.57)
Solomon Islands	55.7 (36.9, 78.8)	36 (23.7, 51.2)	67.1 (44.1, 94.5)	25.9 (17, 36.6)	-1.1 (-1.16, -1.04)
Somalia	7436.9 (5274.5, 10235.4)	183.1 (128.4, 254)	11810.8 (8205.7, 16267.3)	110.1 (75.8, 153.1)	-1.59 (-1.76, -1.42)
South Africa	51499 (37593.7, 67339)	375.2 (273.5, 491.2)	25630.7 (16939.5, 35423)	169.3 (112.6, 233.1)	-2.39 (-2.81, -1.96)
South Sudan	4388.3 (3159.6, 5992.4)	160.9 (114.7, 221.6)	4841.6 (3364.7, 6687.4)	111.1 (77, 153.8)	-1.14 (-1.37, -0.9)
Spain	586.1 (391.6, 825.2)	7 (4.8, 9.7)	141.3 (80.7, 223.2)	2.1 (1.2, 3.3)	-4.72 (-5.34, -4.1)
Sri Lanka	934.7 (611, 1374.8)	16.7 (11, 24.4)	455.5 (303.2, 660.5)	8.9 (6, 12.8)	-2.61 (-2.99, -2.23)
Sudan	5864.2 (4010.9, 8440.1)	65.9 (44.6, 95.5)	3101.3 (2072.5, 4457.2)	18.6 (12.5, 26.8)	-4.06 (-4.17, -3.94)

Location	1990		2021		EAPC_95%CI
	Number (95%UI)	ASR (95%UI)	Number (95%UI)	ASR (95%UI)	
Suriname	22.2 (14.9, 31.4)	17.1 (11.6, 24.1)	8.8 (5.9, 12.8)	6.2 (4.2, 8.8)	-3.42 (-3.58, -3.26)
Sweden	41.7 (27.2, 61.9)	2.7 (1.8, 4)	61.4 (37.8, 95.8)	3.3 (2.1, 5.2)	1.18 (1.01, 1.35)
Switzerland	46.3 (29.1, 69.6)	4 (2.5, 6)	28.8 (18.1, 43.4)	2.2 (1.4, 3.2)	-1.86 (-2.17, -1.53)
Syrian Arab Republic	805.4 (538.7, 1146.9)	13.7 (9.1, 19.5)	155.3 (99, 228.7)	3.9 (2.5, 5.7)	-4.02 (-4.45, -3.59)
Taiwan (Province of China)	820.5 (545.7, 1177.6)	15.1 (10.2, 21.4)	333.2 (224.2, 485.8)	11.6 (7.9, 16.7)	-4.85 (-6.38, -3.3)
Tajikistan	1110.7 (767.7, 1537.6)	46.9 (32, 65.6)	620.6 (437.7, 864.6)	17.1 (12, 24)	-3.62 (-3.93, -3.31)
Thailand	8675.4 (5673, 12581.3)	50.7 (33.5, 73.2)	3348.4 (2188.5, 4843.5)	32.7 (21.5, 47)	-1.3 (-1.34, -1.26)
Timor-Leste	580.7 (400.7, 807)	173.5 (118, 243.1)	502.2 (342.7, 698.4)	96 (65.4, 133.6)	-2.35 (-2.66, -2.03)
Togo	3217.6 (2313.8, 4266)	174.7 (124.6, 233.3)	2190.9 (1537.5, 3059.2)	66.1 (46.3, 92.4)	-3.02 (-3.13, -2.91)
Tokelau	0.1 (0.1, 0.2)	21.7 (14.3, 31)	0 (0, 0.1)	10 (6.6, 14.4)	-2.77 (-2.92, -2.61)
Tonga	6.8 (4.5, 10)	16.2 (10.7, 23.9)	4.2 (2.8, 6)	10.8 (7.1, 15.6)	-1.47 (-1.64, -1.3)
Trinidad and Tobago	38.5 (25.3, 56.5)	9.6 (6.3, 14)	8.9 (5.9, 13)	3.3 (2.2, 4.8)	-3.85 (-3.97, -3.73)
Tunisia	1148.6 (770.7, 1635.8)	37 (24.9, 52.7)	220.2 (145.6, 322.2)	8 (5.3, 11.6)	-5.13 (-5.21, -5.05)
Turkey	5508.8 (3749.4, 7854.7)	26.8 (18.3, 38.1)	711.7 (463.7, 1035.9)	3.8 (2.5, 5.5)	-6.83 (-7.02, -6.63)
Turkmenistan	348.3 (245.9, 473.3)	22.6 (15.8, 30.9)	182.7 (119.3, 262.8)	12 (7.8, 17.3)	-3 (-3.44, -2.56)
Tuvalu	5.7 (3.8, 7.9)	167.1 (110, 235.4)	2.8 (1.9, 4.1)	75.2 (50.8, 109.6)	-2.65 (-2.71, -2.59)
Uganda	15041.3 (10157.5, 20360.2)	167.3 (112.1, 228)	15787.3 (10594.4, 22230)	78.4 (52.3, 111)	-2.1 (-2.38, -1.82)
Ukraine	1273.9 (806.6, 1942.8)	11.1 (7.1, 16.9)	561.6 (335.3, 868.9)	8.4 (5.1, 12.8)	-0.62 (-0.98, -0.26)
United Arab Emirates	50.8 (35.9, 69.1)	8.8 (6.2, 11.9)	23.8 (15.8, 34)	1.8 (1.2, 2.6)	-5.03 (-5.27, -4.8)
United Kingdom	742.3 (467.6, 1114.5)	6.8 (4.3, 10.2)	521.9 (316.1, 821.7)	4.3 (2.6, 6.7)	0.32 (-0.49, 1.12)
United Republic of Tanzania	17785.6 (13867.9, 22357.7)	138.7 (107.5, 175.2)	14307.4 (9619, 20299.7)	58.1 (38.9, 82.6)	-3.79 (-4.31, -3.27)
United States of America	405 (251.7, 621.4)	0.7 (0.4, 1.1)	430.7 (276.2, 647.8)	0.7 (0.5, 1.1)	1.52 (0.88, 2.17)
United States Virgin Islands	1.5 (1, 2.1)	4.5 (3.1, 6.5)	0.2 (0.1, 0.2)	1.3 (0.9, 1.9)	-4.15 (-4.26, -4.05)
Uruguay	90.6 (59.8, 133.1)	11 (7.3, 16.2)	43.4 (27.1, 64.1)	6.4 (4, 9.4)	-1.47 (-1.72, -1.21)
Uzbekistan	2597.3 (1740, 3715.7)	30.3 (20.1, 43.7)	1753.1 (1195.4, 2474.5)	17.6 (12, 25)	0.06 (-0.55, 0.66)
Vanuatu	15.5 (10.4, 22.1)	23.1 (15.3, 33.2)	25.6 (17.1, 36)	22.2 (14.8, 31.3)	-0.05 (-0.13, 0.02)
Venezuela (Bolivarian Republic of)	957.3 (646.1, 1353.6)	13.4 (9.1, 19)	335.8 (229.5, 476.1)	5.1 (3.5, 7.1)	-3.34 (-3.63, -3.04)
Viet Nam	18907.3 (12927, 26044.4)	71.5 (48.9, 98.4)	6352 (4375.4, 8833.1)	25.9 (17.9, 35.9)	-3.26 (-3.5, -3.02)
Yemen	2053.7 (1418, 2876.9)	28.8 (19.7, 40.6)	1148.3 (784.9, 1635.1)	8.3 (5.7, 11.8)	-3.84 (-3.97, -3.72)
Zambia	7030 (4775.5, 9752.9)	177.9 (119.6, 249.1)	7888.9 (4876.4, 11610.7)	95.4 (58.9, 140.6)	-3.01 (-3.6, -2.41)
Zimbabwe	11555.6 (7310.4, 17060.8)	238.9 (150.9, 353.1)	9361.5 (5806.7, 13900.9)	148.7 (92.2, 221)	-0.8 (-1.14, -0.46)

Location	1990		2021		EAPC_95%CI
	Number (95%UI)	ASR (95%UI)	Number (95%UI)	ASR (95%UI)	
**Deaths**					
Afghanistan	2414.9 (1645.4, 3418.9)	50.7 (34.4, 72.3)	1202.8 (824.8, 1755.5)	8 (5.5, 11.7)	-5.33 (-5.72, -4.94)
Albania	15.1 (11.4, 19.9)	1.3 (1, 1.7)	0.5 (0.4, 0.8)	0.1 (0.1, 0.2)	-8.02 (-8.43, -7.6)
Algeria	608 (401.8, 920.6)	5.7 (3.8, 8.6)	60.2 (41.2, 91.4)	0.5 (0.3, 0.7)	-7.22 (-7.41, -7.02)
American Samoa	0.1 (0.1, 0.1)	0.4 (0.3, 0.5)	0 (0, 0)	0.1 (0.1, 0.2)	-4.58 (-4.97, -4.2)
Andorra	0 (0, 0)	0 (0, 0)	0 (0, 0)	0 (0, 0)	-6.55 (-7.09, -6.01)
Angola	5254.9 (3555.2, 7092.3)	96.5 (65.2, 130.5)	2033.5 (1244.2, 2895.4)	12.8 (7.8, 18.3)	-6.47 (-7.04, -5.9)
Antigua and Barbuda	0 (0, 0)	0.1 (0.1, 0.1)	0 (0, 0)	0 (0, 0)	-5.49 (-5.84, -5.14)
Argentina	100.7 (92.2, 110.3)	1 (0.9, 1.1)	10.2 (8.5, 12.3)	0.1 (0.1, 0.1)	-7 (-7.39, -6.61)
Armenia	9.9 (8.5, 11.5)	0.9 (0.8, 1.1)	0.8 (0.6, 1)	0.1 (0.1, 0.2)	-6.55 (-7.53, -5.55)
Australia	0.9 (0.8, 1.1)	0 (0, 0)	0.2 (0.2, 0.2)	0 (0, 0)	-5.51 (-5.83, -5.18)
Austria	0.5 (0.5, 0.6)	0 (0, 0)	0.1 (0, 0.1)	0 (0, 0)	-7.28 (-7.54, -7.01)
Azerbaijan	173.3 (139.5, 211.5)	6.8 (5.4, 8.3)	26.2 (17.9, 37.5)	1.2 (0.8, 1.8)	-6.16 (-6.8, -5.51)
Bahamas	0.3 (0.3, 0.4)	0.5 (0.4, 0.6)	0.1 (0, 0.1)	0.1 (0.1, 0.1)	-6.16 (-6.46, -5.85)
Bahrain	0.8 (0.6, 1)	0.5 (0.4, 0.6)	0.2 (0.1, 0.3)	0.1 (0.1, 0.1)	-5.76 (-6.21, -5.31)
Bangladesh	20393 (14084.1, 26729.2)	39.2 (27, 51.4)	1094.9 (729.5, 1524.3)	2.4 (1.6, 3.4)	-8.49 (-8.65, -8.34)
Barbados	0.1 (0.1, 0.1)	0.1 (0.1, 0.2)	0 (0, 0)	0 (0, 0)	-5.72 (-6.09, -5.35)
Belarus	7.1 (5.9, 8.3)	0.3 (0.3, 0.4)	0.8 (0.6, 1.2)	0.1 (0, 0.1)	-5.43 (-6.7, -4.15)
Belgium	0.5 (0.5, 0.6)	0 (0, 0)	0.1 (0.1, 0.1)	0 (0, 0)	-7.17 (-7.44, -6.91)
Belize	2.6 (2.2, 3)	3.1 (2.7, 3.6)	0.3 (0.2, 0.4)	0.3 (0.2, 0.3)	-7.67 (-7.89, -7.45)
Benin	624.3 (436.6, 928.6)	22.7 (15.8, 33.7)	287.4 (148, 662)	4.4 (2.3, 10)	-4.92 (-5.13, -4.72)
Bermuda	0 (0, 0)	0 (0, 0)	0 (0, 0)	0 (0, 0)	-8.1 (-8.57, -7.62)
Bhutan	28.9 (9, 56.3)	10.7 (3.3, 20.9)	2.4 (1.2, 4.4)	1.3 (0.7, 2.4)	-7.3 (-7.74, -6.87)
Bolivia (Plurinational State of)	884.4 (593.6, 1178.5)	31.4 (21.1, 41.9)	67.2 (46.2, 94.1)	1.9 (1.3, 2.7)	-8.87 (-9.01, -8.72)
Bosnia and Herzegovina	4.6 (3.2, 6.3)	0.4 (0.3, 0.6)	0.4 (0.2, 0.5)	0.1 (0.1, 0.1)	-6.38 (-7.04, -5.71)
Botswana	110.8 (67.3, 163)	18.4 (11.2, 27.1)	63.1 (38, 96.6)	9.3 (5.6, 14.2)	-1.63 (-2.02, -1.23)
Brazil	1145 (1008.6, 1307.6)	2.4 (2.1, 2.7)	98.3 (77.1, 121.7)	0.2 (0.2, 0.3)	-8.11 (-8.39, -7.82)
Brunei Darussalam	0.8 (0.6, 1.1)	0.9 (0.7, 1.2)	0.2 (0.1, 0.2)	0.2 (0.1, 0.3)	-4.75 (-4.97, -4.52)
Bulgaria	4.8 (4.4, 5.3)	0.3 (0.3, 0.3)	0.3 (0.2, 0.3)	0 (0, 0)	-8.28 (-9.02, -7.54)
Burkina Faso	1425.1 (996.2, 1913.8)	27 (18.9, 36.3)	1024.7 (616.5, 1726.1)	8.9 (5.4, 15)	-3.05 (-3.25, -2.86)
Burundi	1834.9 (1340.9, 2394.1)	61.2 (44.6, 79.9)	605.1 (330.1, 949.8)	10 (5.4, 15.6)	-5.32 (-5.84, -4.79)

Location	1990		2021		EAPC_95%CI
	Number (95%UI)	ASR (95%UI)	Number (95%UI)	ASR (95%UI)	
Cabo Verde	11 (7.3, 15.8)	6.5 (4.4, 9.4)	0.6 (0.3, 1.4)	0.4 (0.3, 1.1)	-9.72 (-10.37, -9.08)
Cambodia	1997.9 (1079, 2861.4)	40.1 (21.8, 57.4)	204.9 (120, 331.5)	4 (2.4, 6.5)	-7.89 (-8.3, -7.48)
Cameroon	823 (541.4, 1226.3)	14.8 (9.8, 22.1)	635.1 (360, 1226.3)	4.6 (2.6, 8.9)	-3.12 (-3.68, -2.56)
Canada	2.1 (1.9, 2.3)	0 (0, 0)	0.3 (0.2, 0.3)	0 (0, 0)	-6.82 (-7.37, -6.26)
Central African Republic	1597.1 (1107.7, 2110.1)	114.2 (79, 151.3)	1332.4 (867.2, 1921.3)	56.2 (36.6, 81.1)	-2.04 (-2.25, -1.83)
Chad	1170.2 (792.6, 1718.9)	34.1 (23, 50.2)	1579.2 (1058.4, 2282.8)	15.7 (10.5, 22.8)	-2.35 (-2.71, -1.99)
Chile	48.2 (43.5, 52.9)	1.2 (1.1, 1.3)	3.7 (3.1, 4.3)	0.1 (0.1, 0.1)	-6.95 (-7.21, -6.7)
China	20699.4 (17280.4, 24398.7)	6.5 (5.4, 7.6)	310.8 (248.6, 393.1)	0.1 (0.1, 0.2)	-12.18 (-12.6, -11.76)
Colombia	138.9 (118.7, 161.6)	1.2 (1, 1.4)	13.9 (9.9, 19.1)	0.1 (0.1, 0.2)	-7.02 (-7.52, -6.52)
Comoros	70.7 (42.1, 99.7)	30.5 (18.1, 43)	13.7 (8.4, 20.3)	5.8 (3.6, 8.7)	-5.34 (-5.52, -5.16)
Congo	353.4 (220.3, 501.5)	31.8 (19.8, 45.1)	98.9 (56, 149.7)	5.3 (3, 8.1)	-6.07 (-6.68, -5.46)
Cook Islands	0 (0, 0)	0.4 (0.3, 0.5)	0 (0, 0)	0 (0, 0)	-9.14 (-9.47, -8.8)
Costa Rica	4.8 (4.2, 5.4)	0.4 (0.4, 0.5)	0.4 (0.3, 0.4)	0 (0, 0)	-7.53 (-7.93, -7.12)
Coted'Ivoire	1026.5 (734.8, 1525.8)	16 (11.4, 23.8)	571.8 (332.4, 1049.6)	4.7 (2.7, 8.6)	-3.48 (-3.9, -3.05)
Croatia	2.2 (2, 2.5)	0.2 (0.2, 0.3)	0.1 (0.1, 0.2)	0 (0, 0)	-7.83 (-8.19, -7.46)
Cuba	2.2 (2, 2.4)	0.1 (0.1, 0.1)	0.2 (0.1, 0.2)	0 (0, 0)	-6.84 (-7.15, -6.53)
Cyprus	0.1 (0.1, 0.1)	0 (0, 0.1)	0 (0, 0)	0 (0, 0)	-7.91 (-8.54, -7.28)
Czechia	1.5 (1.4, 1.7)	0.1 (0.1, 0.1)	0.1 (0, 0.1)	0 (0, 0)	-9.41 (-9.81, -9)
Democratic People's Republic of Korea	692 (452.4, 1010.1)	10.7 (7, 15.7)	86.2 (53.3, 134.4)	1.9 (1.2, 3)	-5.16 (-5.6, -4.71)
Democratic Republic of the Congo	14673.4 (9789.8, 21686.3)	72.2 (48.1, 106.6)	5347 (3041.4, 9818.2)	13.9 (7.9, 25.5)	-4.65 (-5.33, -3.97)
Denmark	0.2 (0.2, 0.2)	0 (0, 0)	0 (0, 0)	0 (0, 0)	-8.17 (-8.5, -7.84)
Djibouti	44.4 (28.7, 62.1)	24.3 (15.7, 34)	20.7 (11.6, 32)	5 (2.8, 7.7)	-4.99 (-5.67, -4.31)
Dominica	0.2 (0.1, 0.2)	0.7 (0.5, 0.9)	0 (0, 0.1)	0.3 (0.2, 0.5)	-1.81 (-2.4, -1.22)
Dominican Republic	236.4 (188.6, 288)	8.4 (6.7, 10.3)	19 (12.4, 28.6)	0.6 (0.4, 1)	-7.41 (-7.9, -6.92)
Ecuador	224.7 (201.5, 251.7)	5.8 (5.2, 6.5)	14.6 (10.9, 19.3)	0.3 (0.2, 0.4)	-9.37 (-9.65, -9.09)
Egypt	284.9 (219, 367.3)	1.2 (0.9, 1.6)	39.7 (28.5, 53.6)	0.1 (0.1, 0.1)	-6.25 (-6.73, -5.77)
El Salvador	88 (69.8, 110.8)	4 (3.2, 5.1)	3.5 (2.4, 5)	0.2 (0.1, 0.3)	-9.13 (-9.79, -8.46)
Equatorial Guinea	166.6 (112, 226.5)	73.5 (49.3, 100.1)	20 (8.2, 41.5)	3.6 (1.5, 7.5)	-10.85 (-11.37, -10.31)
Eritrea	847.9 (524.5, 1266.6)	48.4 (29.9, 72.3)	351.4 (200.2, 618.3)	13.5 (7.7, 23.8)	-3.93 (-4.11, -3.75)
Estonia	1.2 (1.1, 1.4)	0.4 (0.3, 0.4)	0.1 (0, 0.1)	0 (0, 0)	-7.65 (-8.78, -6.5)
Eswatini	94 (58.6, 135.1)	23.2 (14.5, 33.4)	49.1 (32.2, 73.1)	12.1 (7.9, 18)	-1.5 (-2.22, -0.76)

Location	1990		2021		EAPC_95%CI
	Number (95%UI)	ASR (95%UI)	Number (95%UI)	ASR (95%UI)	
Ethiopia	20491.1 (14922, 26258.3)	75.6 (55, 97)	2583.4 (1855.5, 3432.3)	5.7 (4.1, 7.6)	-8.6 (-8.86, -8.34)
Fiji	3.4 (2.4, 4.8)	1.2 (0.8, 1.7)	1.8 (1.2, 2.5)	0.7 (0.5, 0.9)	-2.29 (-2.71, -1.87)
Finland	0.4 (0.4, 0.5)	0 (0, 0)	0 (0, 0)	0 (0, 0)	-7.14 (-7.45, -6.83)
France	8.2 (7.4, 9)	0.1 (0.1, 0.1)	1 (0.9, 1.2)	0 (0, 0)	-6.89 (-7.25, -6.53)
Gabon	64.6 (36.6, 96.5)	14.8 (8.4, 22.1)	17.7 (8.1, 30.3)	2.9 (1.3, 4.9)	-4.45 (-4.97, -3.92)
Gambia	80.7 (53.7, 126.3)	15.7 (10.4, 24.6)	40.6 (25.4, 72.3)	4 (2.5, 7.1)	-4.43 (-4.79, -4.06)
Georgia	18.6 (15.3, 22.3)	1.4 (1.1, 1.7)	1.3 (1, 1.6)	0.2 (0.1, 0.2)	-7.19 (-8.01, -6.36)
Germany	4.4 (4, 4.9)	0 (0, 0)	0.4 (0.3, 0.5)	0 (0, 0)	-7.18 (-7.4, -6.96)
Ghana	1049 (705.1, 1817.1)	14.3 (9.6, 24.8)	371 (209.2, 890.9)	2.8 (1.6, 6.8)	-4.26 (-4.55, -3.96)
Greece	0.7 (0.6, 0.8)	0 (0, 0)	0.1 (0.1, 0.1)	0 (0, 0)	-5.76 (-6.13, -5.39)
Greenland	0.2 (0.1, 0.3)	1.3 (0.9, 1.7)	0 (0, 0)	0.1 (0.1, 0.2)	-7.49 (-7.74, -7.23)
Grenada	0.1 (0.1, 0.1)	0.2 (0.2, 0.3)	0 (0, 0)	0 (0, 0)	-6.45 (-6.75, -6.16)
Guam	0.3 (0.2, 0.4)	0.7 (0.6, 0.9)	0.1 (0.1, 0.2)	0.3 (0.2, 0.4)	-2.75 (-3.22, -2.27)
Guatemala	315.8 (277.3, 358.2)	7.5 (6.5, 8.5)	11.5 (9.1, 14.6)	0.2 (0.2, 0.3)	-10.67 (-10.96, -10.38)
Guinea	1080.3 (758.7, 1467.4)	33.5 (23.5, 45.5)	405 (233.6, 738.8)	6.4 (3.7, 11.7)	-4.48 (-4.8, -4.16)
Guinea-Bissau	222.7 (150.4, 312.6)	42.4 (28.6, 59.6)	68.2 (40.6, 138.6)	7.3 (4.4, 14.9)	-5.13 (-5.68, -4.57)
Guyana	10.8 (8.5, 13.4)	3.5 (2.8, 4.4)	0.9 (0.7, 1.2)	0.4 (0.3, 0.6)	-5.02 (-5.54, -4.5)
Haiti	741.3 (555.3, 1070.9)	25.1 (18.7, 36.8)	246.3 (156.1, 424.6)	5.6 (3.5, 9.6)	-4.52 (-4.81, -4.23)
Honduras	102.2 (71.3, 136.3)	4.5 (3.2, 6)	9.3 (5, 15.2)	0.3 (0.2, 0.5)	-8.84 (-8.97, -8.71)
Hungary	4.5 (4.1, 5)	0.2 (0.2, 0.3)	0.2 (0.2, 0.3)	0 (0, 0)	-8.44 (-8.82, -8.06)
Iceland	0 (0, 0)	0 (0, 0)	0 (0, 0)	0 (0, 0)	-7.17 (-7.41, -6.92)
India	55562.1 (43539.5, 69840.8)	16.8 (13.2, 21.1)	7792.6 (5889.9, 10408.6)	2.3 (1.7, 3)	-6.46 (-6.7, -6.22)
Indonesia	14934.9 (11437.4, 18618.8)	22.9 (17.5, 28.5)	2199.3 (1678.6, 2823.1)	3.4 (2.6, 4.4)	-5.9 (-6.06, -5.74)
Iran (Islamic Republic of)	318.7 (230.6, 410.8)	1.3 (0.9, 1.6)	16.4 (13.1, 20.5)	0.1 (0.1, 0.1)	-7.3 (-7.94, -6.66)
Iraq	238.2 (167.7, 327.6)	2.8 (2, 3.8)	26 (18.3, 36.5)	0.2 (0.1, 0.3)	-8.21 (-8.73, -7.69)
Ireland	0.4 (0.3, 0.4)	0 (0, 0)	0 (0, 0)	0 (0, 0)	-8.05 (-8.43, -7.68)
Israel	0.6 (0.5, 0.6)	0 (0, 0)	0.1 (0.1, 0.1)	0 (0, 0)	-8.09 (-8.22, -7.96)
Italy	3.3 (3.1, 3.5)	0 (0, 0)	0.4 (0.3, 0.4)	0 (0, 0)	-7 (-7.39, -6.6)
Jamaica	2.2 (1.9, 2.6)	0.3 (0.2, 0.3)	0.2 (0.1, 0.2)	0 (0, 0)	-6.98 (-7.43, -6.54)
Japan	7.5 (7.2, 7.8)	0 (0, 0)	1 (0.9, 1.1)	0 (0, 0)	-5.79 (-6.03, -5.55)
Jordan	4.8 (3.4, 6.3)	0.3 (0.2, 0.4)	1.6 (1.2, 2.2)	0 (0, 0.1)	-6.37 (-6.76, -5.98)

Location	1990		2021		EAPC_95%CI
	Number (95%UI)	ASR (95%UI)	Number (95%UI)	ASR (95%UI)	
Kazakhstan	167.7 (140.8, 197.2)	3.1 (2.6, 3.7)	20 (15.9, 25.2)	0.4 (0.3, 0.5)	-8.36 (-9.69, -7.01)
Kenya	2666.8 (1273.4, 4448.9)	22 (10.5, 36.8)	902.6 (504.8, 1321.7)	5.2 (2.9, 7.6)	-3.88 (-4.24, -3.53)
Kiribati	10.3 (7.7, 13.5)	32.7 (24.4, 43.1)	4.2 (2.7, 6.2)	10 (6.5, 15)	-3.68 (-3.83, -3.53)
Kuwait	2.3 (2, 2.6)	0.4 (0.3, 0.5)	0.7 (0.6, 0.8)	0.1 (0.1, 0.1)	-4.65 (-5.12, -4.19)
Kyrgyzstan	72.6 (61.2, 85.4)	4 (3.4, 4.7)	17.8 (14.8, 21.2)	0.8 (0.7, 0.9)	-5.79 (-6.78, -4.79)
Lao People's Democratic Republic	846.2 (500.2, 1191.1)	43.1 (25.5, 60.7)	103.9 (61.9, 159.5)	4.4 (2.7, 6.8)	-7.37 (-7.64, -7.1)
Latvia	2.1 (1.9, 2.3)	0.4 (0.3, 0.4)	0.1 (0.1, 0.2)	0 (0, 0.1)	-6.98 (-8.1, -5.84)
Lebanon	5.1 (2.8, 7.8)	0.5 (0.3, 0.7)	0.4 (0.3, 0.6)	0 (0, 0)	-9.01 (-9.3, -8.73)
Lesotho	173.7 (101.8, 267.3)	25 (14.7, 38.5)	139.5 (86.1, 206.5)	23 (14.2, 34.1)	0.31 (-0.08, 0.69)
Liberia	515.7 (355.3, 703.3)	39.6 (27.2, 54)	73.7 (39.3, 148.7)	3.4 (1.8, 6.8)	-8.99 (-9.63, -8.34)
Libya	19.8 (13.5, 26.9)	1.1 (0.7, 1.5)	5.7 (3.3, 8.7)	0.4 (0.3, 0.7)	-1.94 (-2.8, -1.08)
Lithuania	4.1 (3.6, 4.5)	0.5 (0.4, 0.5)	0.2 (0.2, 0.3)	0.1 (0, 0.1)	-6.5 (-7.76, -5.23)
Luxembourg	0 (0, 0)	0 (0, 0)	0 (0, 0)	0 (0, 0)	-7.4 (-8, -6.8)
Madagascar	2553.2 (1799, 3448.8)	42.4 (29.8, 57.3)	922.2 (570.3, 1459.1)	7.9 (4.9, 12.5)	-4.69 (-4.94, -4.43)
Malawi	3552.6 (2435.9, 4918.8)	66.5 (45.6, 92.2)	537 (329, 859.9)	6.8 (4.2, 10.9)	-7.11 (-7.42, -6.8)
Malaysia	100.9 (68.8, 134.2)	1.5 (1, 2)	14.6 (11, 19)	0.2 (0.1, 0.2)	-6.53 (-7.07, -6)
Maldives	14.5 (9.6, 19.5)	13.1 (8.7, 17.6)	0.4 (0.3, 0.5)	0.4 (0.3, 0.6)	-10.61 (-11.18, -10.04)
Mali	1290.2 (885.1, 1828.8)	26.8 (18.4, 38.1)	588 (349.5, 1090.3)	4.6 (2.8, 8.5)	-5.29 (-5.47, -5.12)
Malta	0 (0, 0)	0 (0, 0)	0 (0, 0)	0 (0, 0)	-5.16 (-5.48, -4.83)
Marshall Islands	2.2 (1.4, 3)	10 (6.3, 13.7)	0.9 (0.5, 1.3)	5.2 (3.1, 7.4)	-2.34 (-2.94, -1.73)
Mauritania	124 (85.3, 185.9)	12.1 (8.3, 18.2)	31.4 (20.4, 54.6)	1.7 (1.1, 2.9)	-5.99 (-6.48, -5.5)
Mauritius	0.7 (0.6, 0.8)	0.2 (0.2, 0.3)	0.1 (0.1, 0.1)	0 (0, 0)	-5.72 (-6.12, -5.31)
Mexico	551 (500.1, 615.1)	1.6 (1.5, 1.8)	45 (36, 56.3)	0.1 (0.1, 0.2)	-8.56 (-9.58, -7.52)
Micronesia (Federated States of)	1.7 (1.2, 2.4)	3.8 (2.5, 5.2)	0.2 (0.2, 0.3)	0.8 (0.5, 1.1)	-5.11 (-5.2, -5.02)
Monaco	0 (0, 0)	0.1 (0, 0.1)	0 (0, 0)	0 (0, 0)	-6.52 (-6.79, -6.26)
Mongolia	211.7 (145.5, 294.2)	22 (15.1, 30.6)	18 (11.8, 27.5)	1.6 (1.1, 2.5)	-8.57 (-9.05, -8.08)
Montenegro	0.3 (0.2, 0.4)	0.2 (0.1, 0.3)	0 (0, 0)	0 (0, 0)	-7.73 (-8.43, -7.02)
Morocco	920.9 (663.1, 1275.1)	9.2 (6.6, 12.7)	66.4 (42.2, 107.1)	0.7 (0.4, 1.1)	-7.71 (-8.01, -7.41)
Mozambique	6792.3 (4501.6, 9821.8)	99.5 (65.9, 143.9)	2088 (1386.2, 2926.6)	14.2 (9.4, 19.9)	-5.63 (-5.89, -5.36)
Myanmar	7335 (3948.3, 10278.6)	50.5 (27.1, 70.7)	658.7 (407.2, 978.2)	4.3 (2.7, 6.4)	-8.21 (-8.74, -7.67)
Namibia	123 (80.8, 176.7)	19.6 (12.8, 28.1)	59.1 (37.7, 91.2)	7.3 (4.7, 11.3)	-2.58 (-2.96, -2.21)

Location	1990		2021		EAPC_95%CI
	Number (95%UI)	ASR (95%UI)	Number (95%UI)	ASR (95%UI)	
Nauru	0.2 (0.1, 0.3)	5.7 (3.2, 7.9)	0.1 (0.1, 0.2)	2.7 (1.7, 3.9)	-2.26 (-3.36, -1.14)
Nepal	2215.8 (1580.7, 3133.6)	24.4 (17.4, 34.6)	117.5 (74.7, 170.2)	1.3 (0.8, 1.9)	-9.06 (-9.23, -8.89)
Netherlands	0.8 (0.7, 0.9)	0 (0, 0)	0.1 (0.1, 0.1)	0 (0, 0)	-7.37 (-7.64, -7.1)
New Zealand	0.5 (0.4, 0.6)	0.1 (0.1, 0.1)	0 (0, 0)	0 (0, 0)	-8.2 (-8.81, -7.59)
Nicaragua	122.6 (96.6, 150)	6.5 (5.2, 8)	6.8 (4.7, 9.4)	0.4 (0.2, 0.5)	-9.34 (-9.64, -9.05)
Niger	3242.3 (2274.7, 4860.5)	68.7 (48.1, 103.1)	1635.9 (934.2, 3239.7)	11.6 (6.6, 22.9)	-6.04 (-6.47, -5.61)
Nigeria	18730 (12443.1, 24781.9)	42.1 (28, 55.8)	11585 (6728.9, 17582.7)	11 (6.4, 16.7)	-4.3 (-4.66, -3.93)
Niue	0 (0, 0)	1.2 (0.8, 1.6)	0 (0, 0)	0.5 (0.4, 0.7)	-2.81 (-3.12, -2.49)
North Macedonia	8.2 (6.6, 10.3)	1.7 (1.4, 2.1)	0.1 (0.1, 0.2)	0 (0, 0.1)	-11.35 (-11.98, -10.72)
Northern Mariana Islands	0.1 (0.1, 0.2)	1.1 (0.7, 1.5)	0 (0, 0)	0.3 (0.2, 0.4)	-3.72 (-4.1, -3.34)
Norway	0.1 (0.1, 0.1)	0 (0, 0)	0 (0, 0)	0 (0, 0)	-6.57 (-7.08, -6.06)
Oman	6.2 (4, 9.8)	0.7 (0.5, 1.1)	0.5 (0.4, 0.8)	0 (0, 0.1)	-7.02 (-7.92, -6.11)
Pakistan	12727.1 (8607.3, 18900.3)	24.6 (16.6, 36.6)	6627.7 (4738.1, 8828.1)	7.8 (5.6, 10.4)	-3.21 (-3.4, -3.01)
Palau	0.1 (0, 0.1)	1.3 (0.9, 1.8)	0 (0, 0)	0.4 (0.3, 0.5)	-3.22 (-3.53, -2.9)
Palestine	8.4 (5.7, 12.1)	0.8 (0.5, 1.1)	1.4 (0.9, 2.4)	0.1 (0.1, 0.1)	-6.36 (-6.74, -5.97)
Panama	22.6 (18.9, 26.4)	2.7 (2.3, 3.2)	4 (3.1, 5.1)	0.4 (0.3, 0.5)	-6.23 (-6.73, -5.74)
Papua New Guinea	225.2 (119.9, 373.9)	12.7 (6.8, 21.1)	263.6 (146.7, 401.1)	6.4 (3.6, 9.7)	-1.92 (-2.32, -1.52)
Paraguay	38.4 (28.6, 50.5)	2.2 (1.6, 2.9)	9.6 (6.4, 13.8)	0.5 (0.3, 0.7)	-4.43 (-4.76, -4.1)
Peru	1416.8 (1135.2, 1730.7)	17 (13.6, 20.8)	70.5 (45.7, 102.8)	0.7 (0.5, 1.1)	-10.06 (-10.5, -9.61)
Philippines	3070 (2183.3, 3957)	11.9 (8.5, 15.3)	948.7 (761.3, 1161.9)	2.8 (2.3, 3.5)	-3.74 (-3.99, -3.48)
Poland	5 (4.8, 5.3)	0.1 (0.1, 0.1)	0.2 (0.1, 0.2)	0 (0, 0)	-9.52 (-9.92, -9.13)
Portugal	3.1 (2.7, 3.5)	0.2 (0.1, 0.2)	0.2 (0.1, 0.2)	0 (0, 0)	-8.98 (-9.62, -8.34)
Puerto Rico	1.5 (1.3, 1.7)	0.2 (0.1, 0.2)	0 (0, 0)	0 (0, 0)	-8.65 (-9.06, -8.23)
Qatar	1.5 (1, 2.1)	1.1 (0.8, 1.6)	0.4 (0.3, 0.6)	0.1 (0.1, 0.1)	-7.71 (-7.93, -7.5)
Republic of Korea	172.2 (130.4, 221)	1.6 (1.2, 2)	2.2 (1.6, 2.8)	0 (0, 0)	-11.27 (-11.63, -10.9)
Republic of Moldova	12.1 (10.5, 14)	1 (0.9, 1.1)	0.7 (0.5, 0.9)	0.2 (0.1, 0.2)	-6 (-6.59, -5.41)
Romania	93.9 (85.5, 102.8)	1.8 (1.7, 2)	10.2 (8.3, 12.3)	0.4 (0.3, 0.5)	-5.89 (-6.54, -5.24)
Russian Federation	241.4 (230.7, 252.6)	0.7 (0.7, 0.8)	23.5 (20.9, 25.6)	0.1 (0.1, 0.1)	-6.39 (-7.69, -5.08)
Rwanda	1683.4 (952, 2293.3)	44.7 (25.3, 60.9)	210.1 (135.3, 311.9)	4.2 (2.7, 6.3)	-8.65 (-9.37, -7.93)
Saint Kitts and Nevis	0.1 (0.1, 0.1)	0.7 (0.6, 0.8)	0 (0, 0)	0.1 (0.1, 0.1)	-5.33 (-5.82, -4.84)
Saint Lucia	0.4 (0.3, 0.5)	0.8 (0.6, 0.9)	0 (0, 0)	0.1 (0.1, 0.1)	-6.28 (-6.57, -5.99)

Location	1990		2021		EAPC_95%CI
	Number (95%UI)	ASR (95%UI)	Number (95%UI)	ASR (95%UI)	
Saint Vincent and the Grenadines	0.3 (0.3, 0.4)	0.9 (0.7, 1.1)	0 (0, 0)	0.1 (0.1, 0.1)	-7 (-7.27, -6.72)
Samoa	1.3 (0.9, 2)	1.9 (1.2, 2.7)	0.4 (0.2, 0.6)	0.5 (0.3, 0.7)	-4.05 (-4.2, -3.9)
San Marino	0 (0, 0)	0 (0, 0)	0 (0, 0)	0 (0, 0)	-7.83 (-8.31, -7.35)
Sao Tome and Principe	4.5 (3.2, 6.3)	7.8 (5.6, 10.9)	0.3 (0.2, 0.7)	0.4 (0.2, 0.9)	-9.17 (-9.61, -8.71)
Saudi Arabia	214.5 (142.9, 309.8)	3.2 (2.1, 4.6)	6.3 (4.2, 9.7)	0.1 (0.1, 0.1)	-11.23 (-11.31, -11.15)
Senegal	711.4 (526.9, 944)	17.5 (12.9, 23.2)	152.4 (98.5, 296.5)	2.4 (1.5, 4.6)	-6.04 (-6.42, -5.65)
Serbia	10.7 (7.7, 13.9)	0.5 (0.4, 0.7)	0.1 (0.1, 0.2)	0 (0, 0)	-12.38 (-12.97, -11.78)
Seychelles	0.1 (0.1, 0.1)	0.3 (0.3, 0.4)	0 (0, 0)	0.2 (0.1, 0.2)	-2.54 (-2.93, -2.15)
Sierra Leone	993.5 (712.9, 1325.8)	45.9 (32.9, 61.3)	437.5 (289, 699.6)	11.6 (7.6, 18.5)	-4.31 (-4.81, -3.8)
Singapore	2.2 (1.9, 2.5)	0.4 (0.3, 0.4)	0.2 (0.2, 0.2)	0 (0, 0)	-8.02 (-8.33, -7.72)
Slovakia	1.6 (1.3, 1.9)	0.1 (0.1, 0.2)	0.2 (0.1, 0.2)	0 (0, 0)	-6.09 (-6.29, -5.88)
Slovenia	0.4 (0.3, 0.4)	0.1 (0.1, 0.1)	0 (0, 0)	0 (0, 0)	-9.84 (-10.32, -9.37)
Solomon Islands	6.1 (3.8, 9.5)	3.8 (2.3, 5.9)	3.5 (2.3, 5.2)	1.3 (0.9, 2)	-3.07 (-3.29, -2.86)
Somalia	2465 (1413.7, 4358)	56.5 (32.3, 100)	2876.7 (1510.3, 5386.3)	24.9 (13.1, 46.7)	-2.44 (-2.86, -2.03)
South Africa	3285.8 (2538.5, 4495.9)	23.7 (18.3, 32.5)	1220.3 (950.1, 1539.2)	8.3 (6.5, 10.5)	-3.23 (-3.86, -2.6)
South Sudan	1435.4 (876.9, 2172.4)	49.7 (30.4, 75.3)	1297.7 (847.2, 2033)	29.2 (19.1, 45.7)	-1.68 (-2.45, -0.9)
Spain	3.9 (3.4, 4.4)	0.1 (0, 0.1)	0.3 (0.2, 0.3)	0 (0, 0)	-8.02 (-8.18, -7.86)
Sri Lanka	79.6 (60.6, 100.6)	1.5 (1.1, 1.8)	7.4 (5.1, 10.6)	0.1 (0.1, 0.2)	-7.5 (-8.03, -6.97)
Sudan	1270.8 (854.1, 1738.4)	13.1 (8.8, 17.9)	163.2 (100.1, 247.2)	1 (0.6, 1.5)	-7.57 (-7.86, -7.28)
Suriname	1.2 (0.8, 1.6)	0.9 (0.6, 1.2)	0.3 (0.2, 0.4)	0.2 (0.1, 0.3)	-5.38 (-5.73, -5.03)
Sweden	0.2 (0.2, 0.2)	0 (0, 0)	0 (0, 0)	0 (0, 0)	-7 (-7.34, -6.65)
Switzerland	0.3 (0.3, 0.4)	0 (0, 0)	0 (0, 0)	0 (0, 0)	-7.96 (-8.32, -7.61)
Syrian Arab Republic	57.2 (39.4, 80.4)	0.9 (0.7, 1.3)	3.1 (2.1, 4.8)	0.1 (0.1, 0.1)	-7.09 (-8.45, -5.71)
Taiwan (Province of China)	52.7 (47.9, 58.1)	1.1 (1, 1.2)	2 (1.6, 2.4)	0.1 (0.1, 0.1)	-8.86 (-9.16, -8.56)
Tajikistan	340.4 (266, 429.3)	12.8 (10, 16.1)	133.4 (83.1, 200.5)	3.5 (2.2, 5.3)	-4.56 (-4.92, -4.21)
Thailand	506.1 (358.5, 709.3)	3.2 (2.2, 4.4)	48.1 (36.5, 61.8)	0.5 (0.4, 0.7)	-5.32 (-5.59, -5.06)
Timor-Leste	224.8 (137.1, 359.4)	58.8 (36, 94)	46.7 (30.9, 66.8)	8.9 (5.9, 12.7)	-6.51 (-6.94, -6.07)
Togo	341.3 (244.6, 482.8)	17.8 (12.8, 25.2)	153 (88.4, 290.5)	4.6 (2.7, 8.7)	-3.92 (-4.29, -3.54)
Tokelau	0 (0, 0)	1.6 (1, 2.2)	0 (0, 0)	0.3 (0.2, 0.4)	-5.87 (-6.16, -5.57)
Tonga	0.4 (0.3, 0.7)	1 (0.7, 1.6)	0.1 (0.1, 0.2)	0.4 (0.2, 0.5)	-3.24 (-3.63, -2.83)
Trinidad and Tobago	1.5 (1.3, 1.8)	0.4 (0.3, 0.5)	0.1 (0.1, 0.2)	0.1 (0, 0.1)	-5.92 (-6.21, -5.63)

Location	1990		2021		EAPC_95%CI
	Number (95%UI)	ASR (95%UI)	Number (95%UI)	ASR (95%UI)	
Tunisia	65.2 (44, 95.8)	2.1 (1.4, 3.1)	2.9 (1.8, 4.9)	0.1 (0.1, 0.2)	-8.73 (-8.98, -8.48)
Turkey	751.9 (513.8, 1038.9)	3.8 (2.6, 5.2)	10.9 (7.8, 14.9)	0.1 (0, 0.1)	-13.34 (-14, -12.66)
Turkmenistan	108.9 (93, 127.4)	6.6 (5.6, 7.7)	22.1 (17.5, 27.9)	1.4 (1.1, 1.8)	-5.56 (-6.24, -4.88)
Tuvalu	1 (0.7, 1.4)	27 (17.2, 37.4)	0.1 (0.1, 0.1)	2.8 (1.8, 3.8)	-6.6 (-6.88, -6.33)
Uganda	3093.9 (1976.3, 4902)	30.7 (19.6, 48.7)	1435.2 (830.4, 2382.1)	6.9 (4, 11.5)	-4.62 (-5.01, -4.22)
Ukraine	30 (25.4, 35.5)	0.3 (0.2, 0.3)	6.6 (5.3, 8.2)	0.1 (0.1, 0.2)	-1.76 (-2.77, -0.73)
United Arab Emirates	1.8 (0.7, 4.1)	0.3 (0.1, 0.7)	0.3 (0.1, 0.6)	0 (0, 0)	-7.75 (-8.16, -7.35)
United Kingdom	4 (3.8, 4.2)	0 (0, 0)	0.6 (0.5, 0.6)	0 (0, 0)	-5.64 (-5.99, -5.3)
United Republic of Tanzania	5125.7 (3696.7, 6905.7)	37.8 (27.2, 51)	1733.1 (1089.7, 2487.4)	6.9 (4.3, 9.9)	-4.97 (-5.23, -4.71)
United States of America	22.7 (21.9, 23.5)	0 (0, 0)	3.5 (3.2, 3.9)	0 (0, 0)	-5.98 (-6.58, -5.37)
United States Virgin Islands	0.1 (0, 0.1)	0.2 (0.1, 0.2)	0 (0, 0)	0 (0, 0)	-7.01 (-7.26, -6.76)
Uruguay	3.8 (3.5, 4.3)	0.5 (0.4, 0.5)	0.4 (0.3, 0.5)	0.1 (0, 0.1)	-6.83 (-7.06, -6.6)
Uzbekistan	377.8 (331.2, 431.4)	4 (3.5, 4.5)	103.3 (83.9, 126.8)	1 (0.8, 1.2)	-4.33 (-5.01, -3.63)
Vanuatu	1.8 (1, 3.1)	2.6 (1.5, 4.4)	1.3 (0.8, 2.1)	1.1 (0.7, 1.8)	-2.46 (-2.97, -1.96)
Venezuela (Bolivarian Republic of)	84.4 (76.5, 92.4)	1.2 (1.1, 1.3)	15.7 (11.3, 21.4)	0.2 (0.2, 0.3)	-5.73 (-6.75, -4.7)
Viet Nam	2338.1 (1636.8, 3065.7)	8.7 (6.1, 11.5)	181.5 (122.5, 258.7)	0.8 (0.5, 1.1)	-7.43 (-7.57, -7.29)
Yemen	439.7 (264.4, 670)	5.7 (3.4, 8.7)	109.8 (66.9, 167.7)	0.8 (0.5, 1.2)	-6.35 (-6.5, -6.19)
Zambia	2154.1 (1444.4, 2957.3)	50.6 (33.9, 69.5)	452.4 (259.8, 737.2)	5.4 (3.1, 8.8)	-7.05 (-7.61, -6.49)
Zimbabwe	794.7 (611, 989.8)	15.9 (12.2, 19.9)	1069.7 (685.7, 1527.8)	17 (10.9, 24.2)	1.12 (0.61, 1.62)

Values in parentheses for case numbers and age-standardized rates indicate 95% uncertainty intervals (UI) derived from GBD 2021 estimates. Values in parentheses for EAPC indicate 95% confidence intervals (CI). Abbreviations: ASR, age-standardized rate; EAPC, socio-demographic index; UI, uncertainty interval; CI, confidence interval.
