# Supplementary material for: Tailoring Prevention and Control Strategies for Childhood Tuberculosis: From a Global Analysis of Burden Trends and Inequalities Across Three Age Groups (1990–2021) to Prevention and Control Strategies
Source: Trop Med Infect Dis. 2026 May 9;11(5):129. doi: 10.3390/tropicalmed11050129 (PMC13211679; doi:10.3390/tropicalmed11050129)
Supplement: Supplementary file 1 [file tropicalmed-11-00129-s001.zip › Table_S2.pdf]

Table S2: Full list of countries and territories categorized by Socio-demographic Index (SDI) quintiles and GBD geographical regions

Classification	Countries and Territories Included
**Global**	Includes all countries listed in the 21 GBD geographical regions below.
**High SDI**	United States of America, Canada, Japan, Republic of Korea, Singapore, Australia, New Zealand, United Kingdom, Germany, France, Italy, Spain, Nordic countries (Sweden, Norway, etc.), Israel, United Arab Emirates, Qatar, among others.
**High-middle SDI**	China, Russian Federation, Brazil, Argentina, Chile, Mexico, Turkey, Iran, Saudi Arabia, Poland, Romania, Malaysia, Thailand, among others.
**Middle SDI**	South Africa, Indonesia, Viet Nam, Philippines, Colombia, Peru, Egypt, Morocco, Algeria, Iraq, Sri Lanka, among others.
**Low-middle SDI**	India, Bangladesh, Pakistan, Nigeria, Kenya, Ghana, Myanmar, Nepal, Cambodia, Zimbabwe, among others.
**Low SDI**	Afghanistan, Ethiopia, Uganda, United Republic of Tanzania, Mozambique, Madagascar, Mali, Niger, Chad, Democratic Republic of the Congo, Somalia, Yemen, among others.
**High-income North America**	Canada, Greenland, United States of America.
**Andean Latin America**	Bolivia, Ecuador, Peru.
**Tropical Latin America**	Brazil, Paraguay.
**Central Latin America**	Colombia, Costa Rica, El Salvador, Guatemala, Honduras, Mexico, Nicaragua, Panama, Venezuela.
**Southern Latin America**	Argentina, Chile, Uruguay.
**Caribbean**	Antigua and Barbuda, Bahamas, Barbados, Belize, Bermuda, Cuba, Dominica, Dominican Republic, Grenada, Guyana, Haiti, Jamaica, Puerto Rico, Saint Kitts and Nevis, Saint Lucia, Saint Vincent and the Grenadines, Suriname, Trinidad and Tobago, United States Virgin Islands.
**Western Europe**	Andorra, Austria, Belgium, Cyprus, Denmark, Finland, France, Germany, Greece, Iceland, Ireland, Israel, Italy, Luxembourg, Malta, Monaco, Netherlands, Norway, Portugal, San Marino, Spain, Sweden, Switzerland, United Kingdom.
**Central Europe**	Albania, Bosnia and Herzegovina, Bulgaria, Croatia, Czechia, Hungary, Montenegro, North Macedonia, Poland, Romania, Serbia, Slovakia, Slovenia.
**Eastern Europe**	Belarus, Estonia, Latvia, Lithuania, Moldova, Russian Federation, Ukraine.
**High-income Asia Pacific**	Brunei Darussalam, Japan, Republic of Korea, Singapore.
**Central Asia**	Armenia, Azerbaijan, Georgia, Kazakhstan, Kyrgyzstan, Mongolia, Tajikistan, Turkmenistan, Uzbekistan.
**South Asia**	Afghanistan, Bangladesh, Bhutan, India, Nepal, Pakistan.

**Southeast Asia**	Cambodia, Indonesia, Lao People's Democratic Republic, Malaysia, Maldives, Myanmar, Philippines, Sri Lanka, Thailand, Timor-Leste, Viet Nam.
**East Asia**	China, Democratic People's Republic of Korea, Taiwan (Province of China).
**North Africa and Middle East**	Algeria, Bahrain, Egypt, Iran (Islamic Republic of), Iraq, Jordan, Kuwait, Lebanon, Libya, Morocco, Oman, Palestine, Qatar, Saudi Arabia, Sudan, Syrian Arab Republic, Tunisia, Turkey, United Arab Emirates, Yemen.
**Western Sub-Saharan Africa**	Benin, Burkina Faso, Cabo Verde, Cameroon, Chad, Côte d'Ivoire, Gambia, Ghana, Guinea, Guinea-Bissau, Liberia, Mali, Mauritania, Niger, Nigeria, São Tomé and Príncipe, Senegal, Sierra Leone, Togo.
**Eastern Sub-Saharan Africa**	Burundi, Comoros, Djibouti, Eritrea, Ethiopia, Kenya, Madagascar, Malawi, Mozambique, Rwanda, Somalia, South Sudan, United Republic of Tanzania, Uganda, Zambia.
**Central Sub-Saharan Africa**	Angola, Central African Republic, Congo, Democratic Republic of the Congo, Equatorial Guinea, Gabon.
**Southern Sub-Saharan Africa**	Botswana, Eswatini, Lesotho, Namibia, South Africa, Zimbabwe.
**Australasia**	Australia, New Zealand.
**Oceania**	American Samoa, Cook Islands, Fiji, French Polynesia, Guam, Kiribati, Marshall Islands, Micronesia (Federated States of), Nauru, Niue, Northern Mariana Islands, Palau, Papua New Guinea, Samoa, Solomon Islands, Tokelau, Tonga, Tuvalu, Vanuatu.
